# Ethnopharmacology, Phytochemistry, and Pharmacology of the Genus *Glehnia*: A Systematic Review

**DOI:** 10.1155/2019/1253493

**Published:** 2019-12-14

**Authors:** Min Yang, Xue Li, Lei Zhang, Congcong Wang, Mingyue Ji, Jianping Xu, Keyong Zhang, Jicheng Liu, Chunhong Zhang, Minhui Li

**Affiliations:** ^1^Department of Pharmacy, Baotou Medical College, Baotou 014060, China; ^2^Department of Pharmacy, Inner Mongolia Medical University, Hohhot 010110, China; ^3^Department of Pharmacy, Qiqihar Medical University, Qiqihar 161006, China; ^4^Guangxi Key Laboratory of Medicinal Resources Protection and Genetic Improvement, Guangxi Botanical Garden of Medicinal Plants, Nanning 530023, China; ^5^Pharmaceutical Laboratory, Inner Mongolia Institute of Traditional Chinese Medicine, Hohhot 010020, China

## Abstract

*Glehnia littoralis* Fr. Schmidt ex Miq, the sole species in the genus *Glehnia* (Apiaceae), has long been used in traditional Chinese medicine to treat fatigue, weakness, stomach-yin deficiency, lung heat, cough, dry throat, and thirst. Recently, *G. littoralis* has also been incorporated into a wide range of Chinese vegetarian cuisines. Based on the comprehensive information, advances in botany, known uses, phytochemistry, pharmacology, and toxicity of *G. littoralis*, we aim to highlight research gaps and challenges in studying *G. littoralis* as well as to explore its potential use in plant biotechnology. This may provide more efficient therapeutic agents and health products from *G. littoralis*. A literature search of SciFinder, ScienceDirect, Scopus, TPL, Google Scholar, Baidu Scholar, and Web of Science, books, PhD and MSc dissertations, and peer-reviewed papers on *G. littoralis* research was conducted and comprehensively analyzed. We confirmed that the ethnomedical uses of *G. littoralis* have been recorded in China, Japan, and Korea for thousands of years. A phytochemical investigation revealed that the primary active compounds were phenylpropanoids, coumarins, lignanoids, and flavonoids, organic acids and derivatives, terpenoids, polyacetylenes, steroids, nitrogen compounds, and others. Our analysis also confirmed that the extracts of *G. littoralis* possess immunoregulatory, antitumor, anti-inflammatory, hepatoprotective, antioxidant, neuroprotective, antibacterial, antifungal, and analgesic properties. Although further studies are required, there is strong evidence of the antitumor and immunoregulatory potential of *G. littoralis*. Also, more studies are needed to elucidate the mechanisms of action of its active compounds (e.g., falcarinol and panaxydiol) before any clinical studies can be carried out.

## 1. Introduction

The genus *Glehnia* belongs to the Apiaceae family and contains only one species, *Glehnia littoralis* Fr. Schmidt ex Miq. *G. littoralis* is a perennial herb with the property of salt tolerance, which allows it to grow on the seashores of Northern Pacific countries, particularly China, Japan, Korea, the USSR, Canada, and the USA [1].

*G. littoralis* has been used in traditional medicine as tonic, antipyretic, and analgesic for thousands of years [2]. Its dried root, Glehniae Radix, known as *Beishashen* in China, *Hamaboufu* in Japan [3], and *Heabangpoong* in Korea [4], is commonly used to treat respiratory (rhinitis and asthma) and gastrointestinal (gastric ulcer) and autoimmune-related diseases [5]. As a traditional herbal medicine, Glehniae Radix has a rich cultural heritage and is used in traditional healing practices to treat multiple symptoms including cough, fever, bloody phlegm, fatigue, dry throat, and thirst [6, 7]. Previous studies reported that bioactive components of *G. littoralis* such as coumarins and polyacetylenes exhibit antioxidant, antitumor, blood circulation-promoting, immunomodulatory, and antimicrobial properties [2, 8]. Currently, *G. littoralis* is also recognized as a nutritional supplement due to its high nutritional value; for example, in Japan, the sprouting leaves are served as vegetables [4], while in China the roots are added to porridge [9]. As a popular medicinal and functional biomaterial, *G. littoralis* with its strong soil adaptability has been widely cultivated in northern China and Japan in recent decades [10].

At present, although it is very common to use bibliometric methods to conduct literature review of a certain field [11–14], this review provides the available information on *G. littoralis* from the literary resources, including SciFinder, ScienceDirect, Scopus, TPL, Google Scholar, Baidu Scholar, and Web of Science, books, PhD and MSc dissertations, and peer-reviewed papers. The systematic review on *G. littoralis* serves as a comprehensive overview of past and current studies of traditional practices and activities, and we found that all of during the last fifty years (from 1969 to 2019) available information on *G. littoralis* focuses on the botany, phytochemistry, pharmacological activities, clinical application, and cultivation of *G. littoralis*, while there are few research studies on the traditional uses and toxicity. There are seven reviews in Chinese and one review in English on *G. littoralis*, of which the reviews in Chinese are mainly about its phytochemical and pharmacological research, and the review in English is only about the phytochemical research on *G. littoralis* [8, 15–21]. This review is currently the most advanced systematic review on the botany, traditional uses, ethnopharmacology, phytochemistry, pharmacological activities, and toxicity of *Glehnia littoralis* and provides an in-depth analysis to explore its therapeutic potential for improving human health.

## 2. Botany

**Figure 1:**
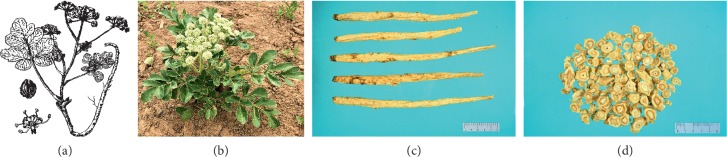
Images of *G. littoralis* from Chinese Materia Medica Dictionary (a), the whole plant of *G. littoralis*, including the flower of *G. littoralis*, fruit of *G. littoralis*, and flowering *G. littoralis* (b), dried roots of *G. littoralis* (c), and sliced roots of *G. littoralis* (d).

*G. littoralis*, a perennial herb, grows 20–70 cm in height. Its root is slender, cylindrical, or spindle-shaped and is yellowish-white in color. The above ground stems are short and branched, whereas the underground part is elongated. The leaves are ovate or oblong, ranging from 1 to 6 cm in length and 0.8 to 3.5 cm in width. Furthermore, they are incised-serrate with white cartilaginous margins and have an obtuse, rounded apex. *G. littoralis* flowers are white, short, and conical. The fruit of *G. littoralis* is double suspended, nearly globose or elliptic, and densely covered with brown spiny soft hairs, with corrugated five fruit ribs that form wing-like structures. The flowering and fruiting period of *G. littoralis* is from June to August [22] (Figure 1).

*G. littoralis* is a cold and drought-resistant plant; however, it thrives in a warm and humid climate. It possesses a strong soil adaptability, and, thus seaside sand or fertile, loose sandy soil is suitable for its cultivation [23]. Currently, *G. littoralis* is widely cultivated in China and Japan. According to the literature, the primary producers of cultivated *G. littoralis* are Shandong Province, Liaoning Province, Hebei Province, Jiangsu Province, Zhejiang Province, Fujian Province, Taiwan, Guangdong Province, and other regions in China. The Laiyang City in Shandong Province is known as the genuine *G. littoralis*-producing area in China where a high-quality herb known as “Laiyang Shashen” is produced in large scale [24]. In recent decades, studies on *G. littoralis* have shown that the production of Laiyang has decreased and that there has been a great effort in finding new places such as Hebei Province and Inner Mongolia to grow the herb. Presently, the Chifeng City in Inner Mongolia and the Anguo City in Hebei Province are the primary production areas of *G. littoralis*, with the Chifeng City being the largest producer [25].

## 3. Traditional Uses and Ethnopharmacology

In ancient China, two medicinal herbs, *Nanshashen* (originated from *Adenophora stricta* Miq.) and *Beishashen* (originated from *G. littoralis*), were referred to as *Shashen* as they had not been distinguished for application purposes. *Shashen* was first recorded in the *Shennong Bencao Jing* (Han Dynasty, 300 AD) [26], and records of the *Beishashen* use in Chinese medicine first appeared in *Benjing Fengyuan* (Qing Dynasty, 1695 AD) [27]. Many other ancient medical books such as *Xinxiu Bencao* (Qing Dynasty, 1757 AD) [28] and *Compendium of Materia Medica* (Qing Dynasty, 1590 AD) [29] include *Beishashen* and described its various therapeutic effects including nourishing yin, erasing lung heat, improving stomach conditions, promoting body fluid production, tonifying deficiency, and reducing fever, chronic bronchitis, tuberculosis, fatigue, dry throat, skin pruritus, restlessness, sleepiness, carbuncle, swelling, and colic. Currently, *Shashen* is only originated from *A. stricta*, which is referred to as *Nanshashen* to distinguish between the two.

*G. littoralis* has been widely used in traditional Chinese medicine (TCM). The root is often used as a drug in clinics to invigorate yin. When dried, the roots are sweet, slightly bitter, and slightly cold and are used to nourish yin, moisten the lung, expel phlegm, and prevent cough [6]. In addition to being used in TCM, *G. littoralis* is also used in ethnic medicine. For example, in Mongolian medicine, its dried root is mainly used to treat cough induced by lung heat, as well as fever, body fluid deficiency, thirst, and other conditions, while in Tibetan medicine, the root is primarily used to treat rheumatism, paralysis, and skin diseases, among others [30]. *G. littoralis* is also used globally owing to its different therapeutic effects. In Japan, it is mainly used to relieve pain, reduce fever, and mitigate or eliminate phlegm, while in Korea it is used primarily to treat migraines and headaches [3, 31].

The dried root of *G. littoralis* is often combined with other Chinese medicinal materials such as Ophiopogonis Radix, Rehmanniae Radix, Mori Folium, Forsythiae Fructus, Lycii Fructus, and Scrophulariae Radix in various complex prescription formulas such as Pinggan Yangfei Decoction, Shashen Maidong Decoction, and Yiguanjian Decoction (Table 1). Among them, Yiguanjian Decoction is an ancient prescription used to treat liver diseases [32, 33]. Shashen Maidong Decoction is commonly used in modern times, mainly to nourish yin and treat lung diseases [34, 35]. In addition, there is also a prescription used by the Mongolian people in China, called Chagansaorilao-4 Decoction, to treat cough in children [36]. By combining *G. littoralis* and different Chinese medicinal herbs, the efficacy of each component in the mixture is thought to be enhanced. However, it is noteworthy to mention that the combined use of *G. littoralis* and *V. nigrum* may be toxic and must be used with caution [37].

All the crude drug names in column 2 were identified properly according to Chinese Pharmacopoeia 2015, and the Latin names of the original plants were identified with TPL (http://www.theplantlist.org).

## 4. Phytochemistry

According to the literature, the chemical composition isolated from *G. littoralis* consists primarily of phenylpropanoids, coumarins, lignans, flavonoids, organic acids, terpenoids, polyacetylenes, and steroids. In addition, *G. littoralis* also contains volatile oils, polysaccharides, and polyols. National scholars generally used high-performance liquid chromatography (HPLC), gas chromatography-mass spectrometry (GC-MS), ^13^C-magnetic resonance, hydrogen-magnetic resonance, and column chromatography to separate and identify these complex chemical constituents.  Table 2 shows the chemical constituents found in *G. littoralis*, including chemical composition names and its classes, material distribution, and appropriate citations for this species.

### 4.1. Phenylpropanoids

**Figure 2:**
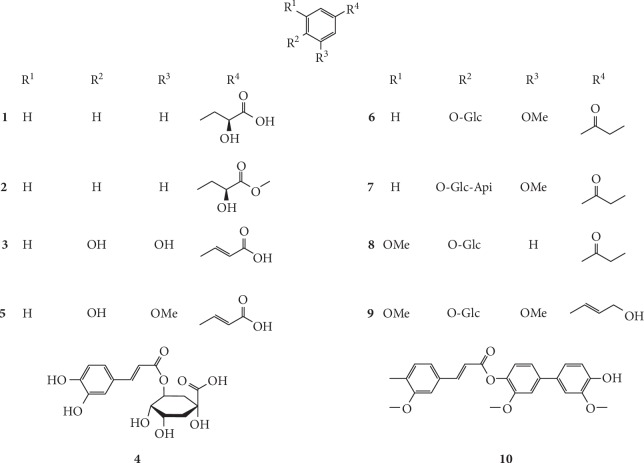
Structures of phenylpropanoids in *G. littoralis.*

Phenylpropanoids widely exist in nature, including most of the natural aromatic compounds. As a major secondary metabolite in *G. littoralis*, phenylpropanoids possess various pharmacological effects including immune regulation, antibacterial, anti-inflammatory, and antioxidative properties. So far, ten phenylpropanoids, including six phenylpropionic acid (**1–5, 10**), three phenylpropanal (**6–8**), and one phenylpropanol (**9**) were isolated from *G. littoralis.* Furthermore, Zhang et al. isolated and identified two simple phenyllactic acid compounds (*S*)-phenyllactic acid (**1**) and (*S*)-phenyllactic acid methyl ester (**2**) via spectral analysis of ethanol extract from the root of *G. littoralis* [19]. It is worth noting that these compounds were isolated from the plant for the second time when studying their taxonomic significance, suggesting that these compounds may be useful chemical taxonomic markers for *G. littoralis*. In addition, Yuan et al. analyzed the ethyl acetate fraction of *G. littoralis* and obtained a phenylpropanoid compound with a unique biphenyl ferulate structure, which was identified through spectroscopy as glehnilate (**10**) [45]. The chemical structures of phenylpropanoids isolated from *G. littoralis* are presented in Figure 2.

### 4.2. Coumarins

Coumarins are the primary components of *G. littoralis* and contain the benzo-*α*-pyrone nucleus. Hydroxyl, alkoxyl, phenyl, and isopentenyl groups, and other substituents are often found attached to the ring [43, 53]. More than 90% of coumarins contain either a hydroxyl or an ether group at C7 position [53, 74]. To date, totally 67 coumarins have been isolated and identified from *G. littoralis*, of which seventeen are simple coumarins (**11–27**), six are pyranocoumarins (**28–33**), and forty-four are furanocoumarins (**34–76**). Among these coumarins, eight new coumarin glycosides, (*S*)-peucedanol 7-*O*-*β*-D-glucopyranoside (**22**), (*S*)-peucedanol 3′-*O*-*β*-D-glucopyranoside (**23**), (*S*)-7-*O*-methylpeucedanol 3′-*O*-*β*-D-glucopyranoside (**25**), (*S*)-peucedanol 3′-*O-β*-D-apiofuranosyl-(1⟶6)-*β*-D-glucopyranoside (**26**), 7-*O*-methylpeucedanol 3′-*O*-*β*-D-apiofuranosyl-(1⟶6)-*β*-D-glucopyranoside (**27**), marmesin 4′-*O-β-*D-apiofuranosyl-(1⟶6)-*β-*D-glucopyranoside (**36**), 4″-hydroxyrmyperatorin 4″-*O-β-*D-glucopyranoside (**59**), and 5″-hydroxyimperatorin 5″-O-*β*-D-glucopyranoside (**60**), were isolated from the methanolic extract of the root and rhizome of *G. littoralis* by Kitajima et al. [53]. A new dihydropyranocoumarin, (+)-*cis*-(3′*S*, 4′*S*)-diisobutyrylkhellactone (**28**), was also isolated from the methanolic extract of the whole plant of *G. littoralis*, and its chemical structure was successfully identified by spectral data interpretation, especially 1D and 2D NMR data [54].

**Figure 3:**
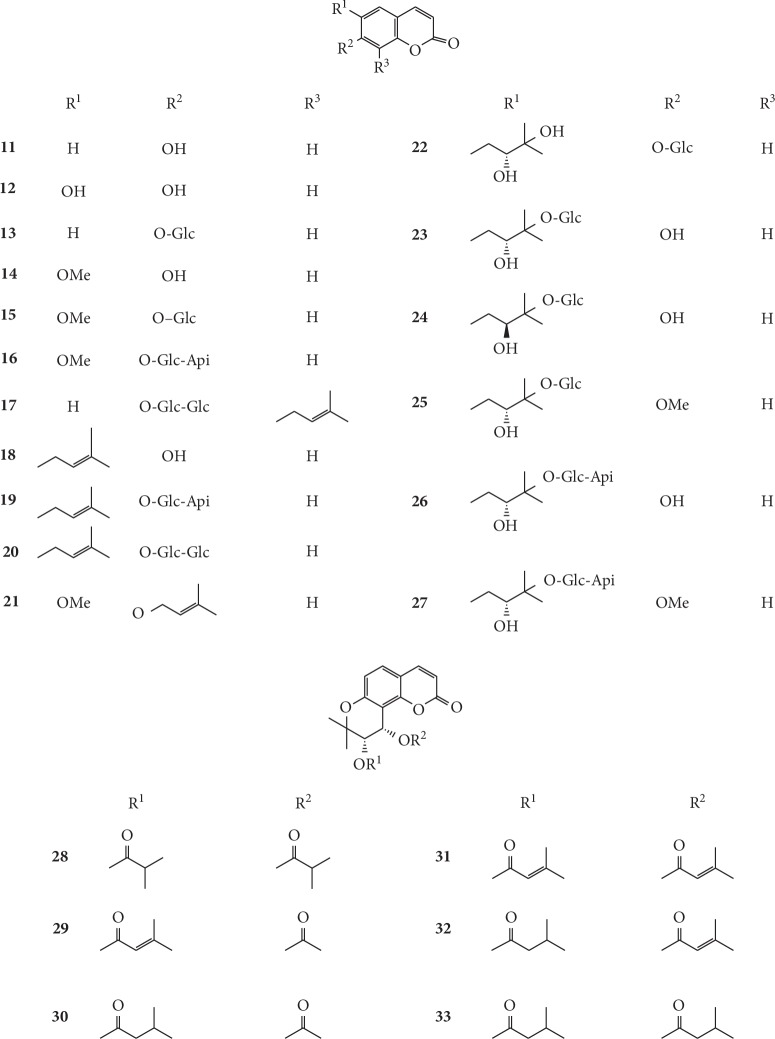
Structures of simple coumarins (**11–27**) and pyranocoumarins (**28–33**) in *G. littoralis.*

**Figure 4:**
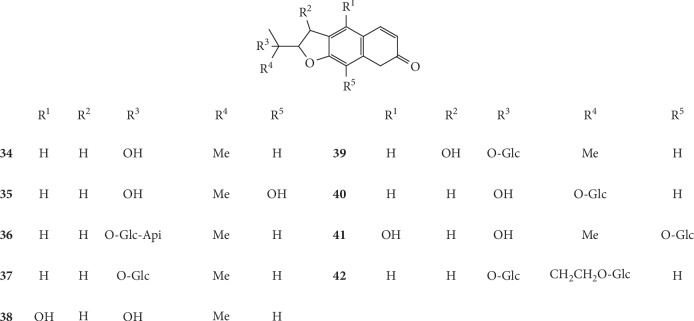
Structures of furanocoumarins in *G. littoralis.*

**Figure 5:**
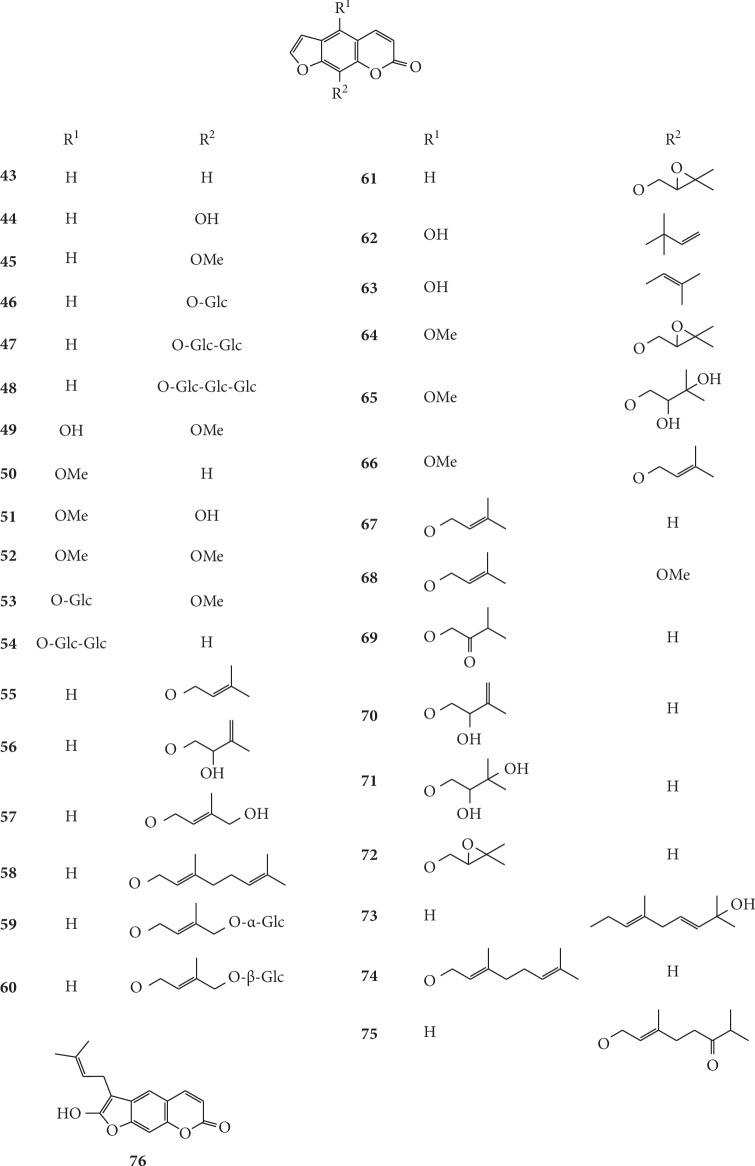
Structures of furanocoumarins in *G. littoralis.*

Currently, the research on coumarins in *G. littoralis* primarily focuses on the determination of coumarins in different parts, on different harvesting dates, and by different treatment methods of *G. littoralis*. In a study on coumarins in *G. littoralis*, Liu et al. determined the total amount of psoralen (**43**), imperatorin (**55**), and isoimperatorin (**67**) in fruits, leaves, roots, and root bark of *G. littoralis*. The results showed that the total content of these three coumarins was the highest in fruit (0.6364 mg·g^−1^), which was 8.24 times higher than that of roots and 42.15 times higher than that of leaves [75]. In another study, Xin et al. (2009) compared the coumarin content in the roots of *G. littoralis* in four different harvesting periods (September 15, September 30, October 15, and October 30 in 2008) and found that the coumarin content (0.0772 mg·g^−1^) and yield (1.0878 mg·strain^−1^) of *G. littoralis* were the highest on October 15 in 2008, which provided important insights to ensure the most effective harvesting of *G. littoralis* [76]. The chemical structures of coumarins isolated from *G. littoralis* are presented in Figures 3–5.

### 4.3. Lignanoids

Lignanoids are natural compounds made from two C6-C3 units. These compounds primarily exist in the wood and resins of plants and are mainly composed of four monomers: cinnamic acid, cinnamyl alcohol, propenyl benzene, and allyl benzene.

**Figure 6:**
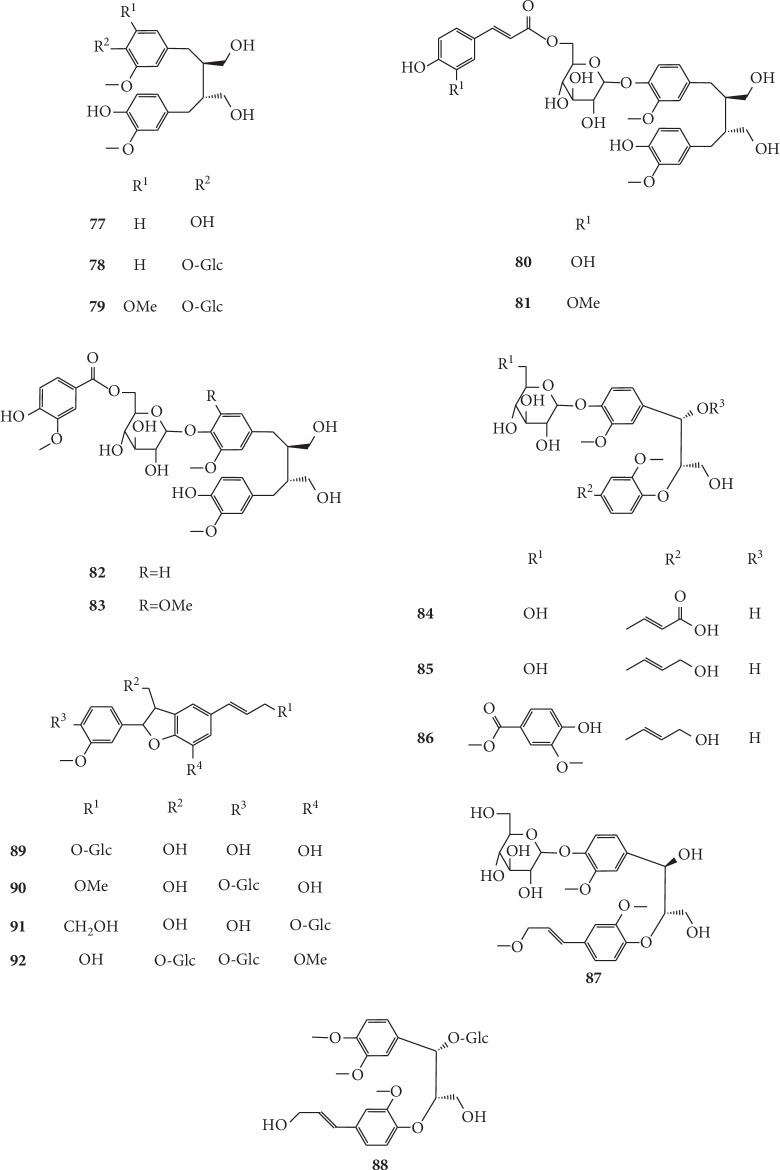
Structures of lignanoids in *G. littoralis.*

There are 16 lignanoids (**77–92**) that have been isolated from *G. littoralis*. Yuan et al. obtained six lignanoids from the underground parts of *G. littoralis*, including (−)-secoisolariciresinol (**77**), (−)-secoisolariciresinol 4-*O*-*β*-D-glucopyranoside (**78**), glehlinosides A (**82**), glehlinoside B (**83**), glehlinoside C (**84**), and citrusin A (**85**). Among these, the compounds **79** and **83–85** were firstly isolated [39]. Xu et al. were the first to isolate four lignanoids from the roots of *G. littoralis*, containing glehlinoside G (**79**), glehlinosides H (**87**), glehlinosides I (**89**), and glehlinoside J (**90**) [64]. Furthermore, two new lignan glycosides, glehlinoside F (**80**) and glehlinoside E (**81**), were isolated by Kong et al. (2008) from the root ethanol extract of *G. littoralis* [61]. In addition, Wang et al. were the first to identify 3-hydroxy-1-(4-hydroxy-3-methoxyphenyl)-2-[4-(3-hydroxy-1-(*E*)-propenyl)-2-methoxyphenoxy] propyl*-β-*D-glucopyranoside (**88**) and 2,3*E*-2,3-dihydro-2-(3′-methoxy-4′-hydroxyphenyl)-3-hydroxymethyl-5-(3″-hydroxypropeyl)-7*-O-β*-D-glucopyranosyl-1-benzo[b] furan (**91**) isolated from *n*-butanol part of 95% ethanol extract of *G. littoralis* [49]. Further, a new 8-*O*-4′ neolignane, glehlinosides D (**86**), was isolated from the dried roots of *G. littoralis* by Wang et al. [63], while a dihydrobenzofuran lignan glycoside, (7*R*,8*S*)-dehydrodiconiferylalcohol-4,9-di-O-*β*-D-glucopyranoside (**92**) was isolated from the same part of *G. littoralis* by Zhao et al. [60]. The chemical structures of lignanoids isolated from *G. littoralis* are presented in Figure 6.

### 4.4. Flavonoids

**Figure 7:**
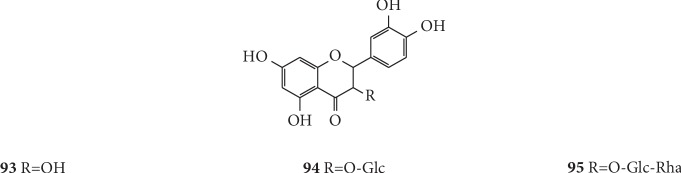
Structures of flavonoids in *G. littoralis.*

There are a total of three flavonoid compounds that have been isolated from *G. littoralis*: quercetin (**93**), isoquercetin (**94**), and rutin (**95**). Yuan et al. successfully isolated and identified 26 compounds from the ethyl acetate fraction of ethanol extract from the underground part of *G. littoralis*, including these three flavonoid compounds [39]. Furthermore, the free radical scavenging test of 1,1-diphenyl-2-picrylhydrazine demonstrated that these three flavonoids were the main antioxidant components in the polar fraction. The chemical structures of flavonoids isolated from *G. littoralis* are presented in Figure 7.

### 4.5. Organic Acids and Derivatives

**Figure 8:**
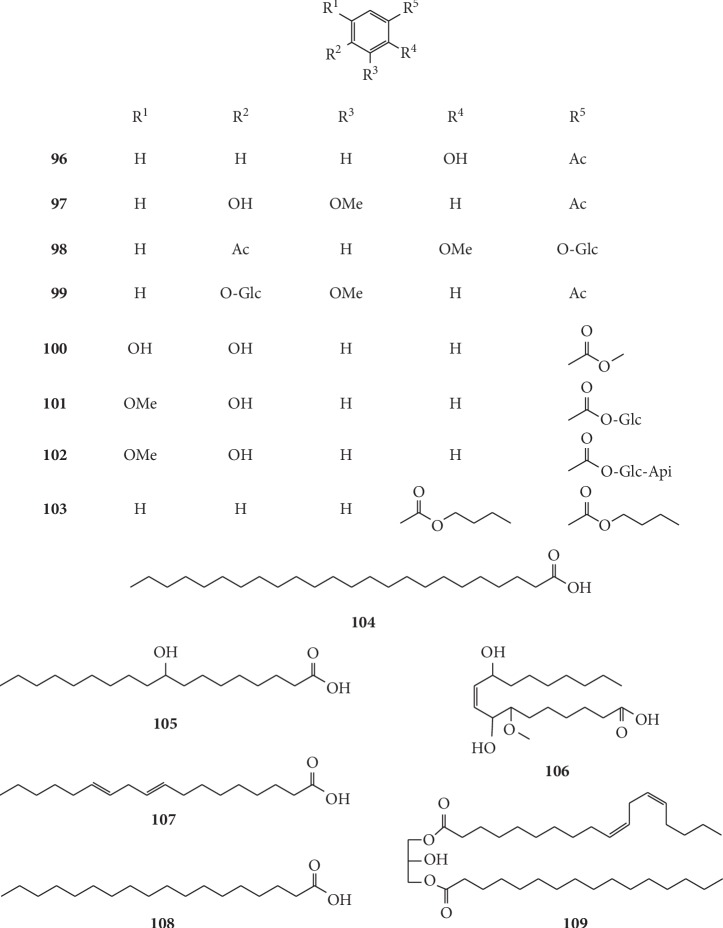
Structures of organic acids and derivatives in *G. littoralis.*

Aromatic acid and fatty acids are common secondary metabolites in many plants. At present, 18 organic acids, existing in *G. littoralis*, have been identified. Yuan et al. isolated and identified two organic acids, namely, salicylic acid (**96**) and vanillic acid (**97**) [40]. Zhang isolated 4-*O-β*-D-glucopyranosyl vanillic acid (**98**), 1-*O*-vanilloyl-*β*-D-glucose (**101**), and a new compound, vanillic acid 1-*O*-[*β*-D-apiofuranosyl-(1 ⟶ 6)-*β*-D-glucopyranoside] ester (**102**) from the *n*-butanol part of the EtOH extract of *G. littoralis*, which were identified by comparing the spectroscopic data (UV, IR, ESI-MS, and ^1^H and ^13^C NMR) [42]. There were an additional nine compounds also isolated from the dried root of *G. littoralis*, including vanillic acid 4-*O-β*-D-glucopyranoside (**99**), protocatechuic acid methyl ester (**100**), dibutyl phthalate (**103**), tetracosanoic acid (**104**), 9-hydroxystearic acid (**105**), glehlinosiden (**106**), linoleic acid (**107**), nonadecanoic acid (**108**), and 1-linoloyl-3-palmitoylglycerol (**109**) [19, 41, 45, 49, 51, 65]. The chemical structures of organic acids and derivatives isolated from *G. littoralis* are presented in Figure 8.

### 4.6. Terpenoids

Terpenoids are products of the mevalonic acid pathway or the deoxyxylulose pathway. An isoprene unit (C5 unit) is the basic structural unit for these compounds and their derivatives. Terpenoids are also important compounds found in *G. littoralis*. Monocyclic monoterpenes and bicyclic monoterpenes are the main terpenoids in *G. littoralis*.

**Figure 9:**
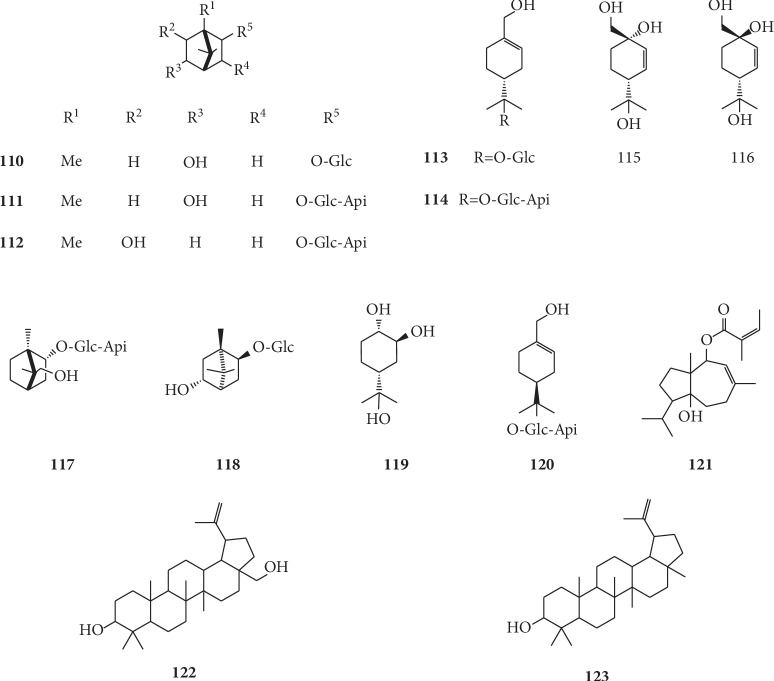
Structures of terpenoids in *G. littoralis.*

Kitajima et al. separated the methanol extracts from the roots and rhizomes of *G. littoralis* using various column chromatography techniques (Sephadex LH-20, silica gel, and Lobar RP-8) and identified them via FAB-MS, ^1^H-NMR, ^13^C-NMR, and HMBC spectrum, allowing for the identification of five monoterpenoids, namely, (-)-angelicoidenol 2-*O*-*β*-D-apiofuranosyl-(1⟶6)-*β*-D-glucopyranoside (**111**), (4*R*)-*p*-menth-1-ene-7,8-diol 8-*O*-*β*-D-apiofuranosyl-(1⟶6)-*β*-D-glucopyranoside (**114**), (2*R*)-bornane-2,9-diol 2-*O*-*β*-D-apiofuranosyl-(1⟶6)-*β*-D-glucopyranoside (**117**), (+)-angelicoidenol [(2*S*,5*R*)-bornane-2,5-diol] 2-*O*-*β*-D-glucopyranoside (**118**), and (4*S*)-*p*-menth-1-ene-7,8-diol 8-*O*-*β*-D-apiofuranosyl-(1⟶6)-*β*-D-glucopyranoside (**120**) [66]. Moreover, Ishikawa et al. (2001) analyzed three monoterpenes and one monoterpene glycoside that were isolated and identified from the water-soluble fraction of the methanol extract of *G. littoralis,* including *trans*-*p*-menth-2-ene-1*α*,2β,8-triol (**119**), *trans*-*p*-menth-2-ene-1,7,8-triol (**115**), *cis*-*p*-menth-2-ene-1,7,8-triol (**116**), and (4*R*)-*p*-menth-1-ene-7,8-diol 8-*O*-*β*-D-glucopyranoside (**113**) [50]. Um et al. isolated (5*β*,10*α*)-lasidiol angelate (**121**) from dried root of *G. littoralis* for the first time and identified its chemical structure using a series of 2D NMR techniques including COSY, HMQC, and HMBC [47]. The chemical structures of terpenoids isolated from *G. littoralis* are presented in Figure 9.

### 4.7. Polyacetylenes

Polyacetylenes are fat-soluble compounds that are abundant in the Apiaceae family and have various biological activities including antibacterial, antifungal, and antitumor. These compounds can be used as important markers to evaluate the quality of *G. littoralis* [19, 67].

**Figure 10:**
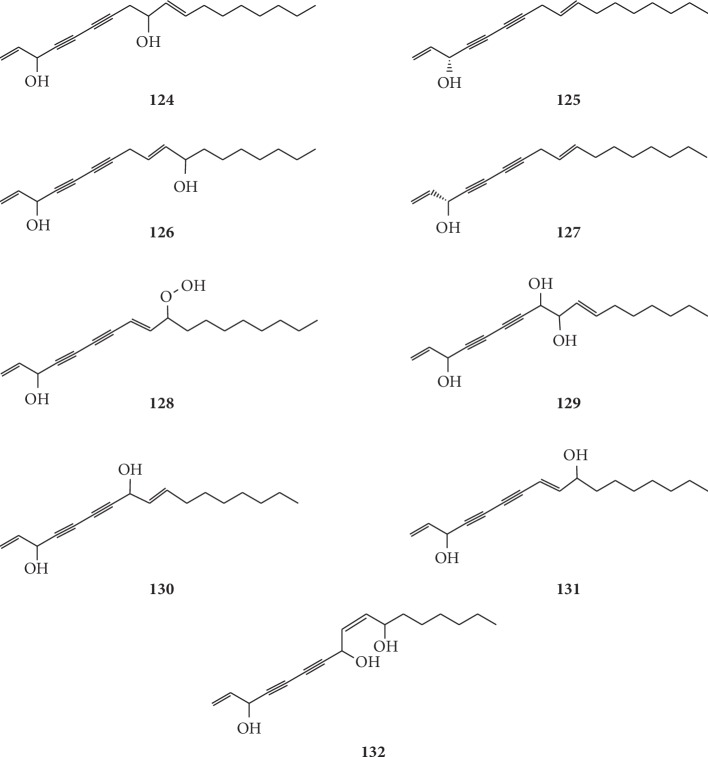
Structures of terpenoids in *G. littoralis.*

Matsuura et al. were the first to isolate two polyacetylene compounds with antibacterial activity from the root of *G. littoralis*, (10*E*)1,10-heptadecadiene-4,6-diyne-3,9-triol (**129**) and (9*Z*)1,9-heptadecadiene-4,6-diyne-3,8,11-triol (**132**) [67]. Further pharmacological analysis revealed strong inhibitory effects against *Escherichia coli*, *Bacillus subtilis*, *Candida albicans*, *Pseudomonas aeruginosa*, and *Staphylococcus aureus*. Su et al. first reported the separation of falcaindiol (**130**) and panaxynol (**131**) from *G. littoralis* using OPLC, which is a planar-layer liquid chromatographic technique that uses external pressure to force the eluent through the sorbent layer [46]. The chemical structures of polyacetylenes isolated from *G. littoralis* are presented in Figure 10.

### 4.8. Steroids

Steroids are nearly ubiquitous in plants and have attracted increasing attention due to their diverse activities. Phytosterols are also important raw materials for the production of steroids and vitamin D3, which are used for the prevention and treatment of coronary atherosclerotic heart disease, and have obvious curative effects on ulcers, skin squamous cell carcinoma, and cervical cancer [59].

**Figure 11:**
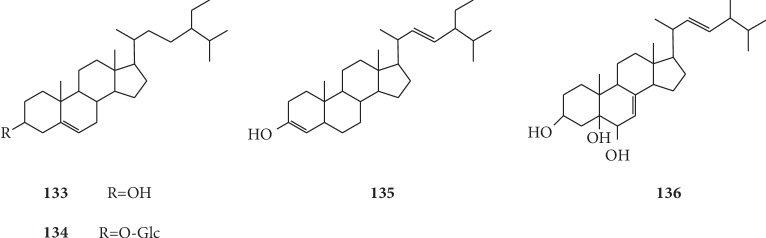
Structures of steroids in *G. littoralis*.

Two steroids were isolated from the petroleum ether fraction of methanol extract of the dried root of *G. littoralis* by Zhang et al.: *β*-sitosterol (**133**) and daucosterol (**134**). And they found that compound **134** was obtained from *G. littoralis* for the first time [59]. Dong et al. also isolated two steroids, stigmasterol (**135**) and cerevisterol (**136**) from dried roots of three-year-old *G. littoralis*, and identified their structure by ^1^H-NMR and ^13^C-NMR [65]. Further, through 3-(4,5-dimethylthiazol-2-yl)-2,5-diphenyltetrazolium bromide (MTT) assays, they determined that compound **135** is capable of inhibiting SGC-7901 and HEP-G2 *in vivo*, and compound **136** was firstly isolated from *G. littoralis*. The chemical structures of steroids isolated from *G. littoralis* are presented in Figure 11.

### 4.9. Nitrogen Compounds

**Figure 12:**
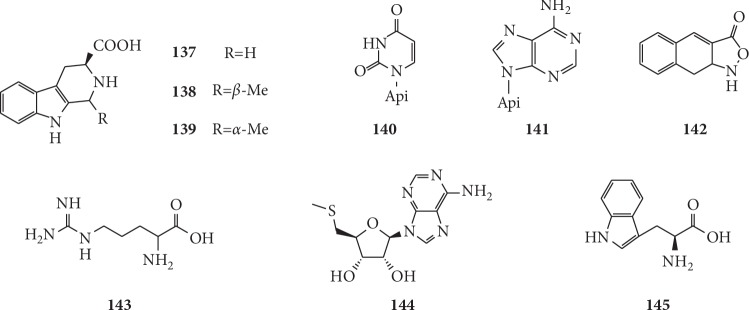
Structures of nitrogen compounds in *G. littoralis.*

Nitrogen compounds are indispensable to living organisms. In recent years, researchers have studied nine nitrogen compounds in *G. littoralis*. Their structures are shown in Figure 12.

It is reported that three *β*-carboline alkaloids, namely, (3*S*)-1,2,3,4-tetrahydro-*β*-carboline-3-carboxylic acid (**137**), (1*S*,3*S*)-1-methyl-1,2,3,4-tetrahydro-*β*-carboline-3-carboxylic acid (**138**), and (1*R*,3*S*)-1-methyl-1,2,3,4-tetrahydro-*β*-carboline-3-carboxylic acid (**139**), were isolated from the ethanolic extract of dried roots of *G. littoralis* by Zhang et al. [19]. The EtOAc fraction of the extraction from *G. littoralis* was chromatographed on a silica gel column to give two nitrogen compounds including uridine (**140**) and adenosine (**141**), which were identified by means of comparison with published data or with authentic samples [39]. Furthermore, Zhao and Yuan isolated 5′-methylthioadenosine (**144**) and L-tryptophan (**145**) from *G. littoralis* by the method of Sephadex LH-20, and the compound **144** was firstly obtained from *G. littoralis* [72].

### 4.10. Other Chemical Constituents

In addition to the above compounds, monosaccharides, polysaccharides, volatile oil amino acid, and trace elements were also found in *G. littoralis.* To examine the relationship between the chemical constituents of *G. littoralis* and its nutritional and health functions, Huang et al. determined the contents of soluble sugar, starch, water-soluble crude polysaccharide, soluble protein, and various amino acids in *G. littoralis.* The contents of chemical substances in *G. littoralis* were high. The contents of soluble sugar, starch, water-soluble crude polysaccharide, and soluble protein were 14.96%, 22.07%, 24.49%, and 3.63%, respectively [71].

Wang et al. used GC-MS to analyze the volatile oils in the roots of *G. littoralis* for the first time. Ten kinds of volatile oils (aldehydes, alcohols, and terpenoids, etc.) were identified and analyzed by normalization method. According to the relative peak area, it was found that the main components were (2E,4E)-deca-2,4-dienal (21.27%) (**172**), followed by (E)-oct-2-en-1-ol (8.53%) (**173**) and **130** (8.15%) [73].

**Figure 13:**
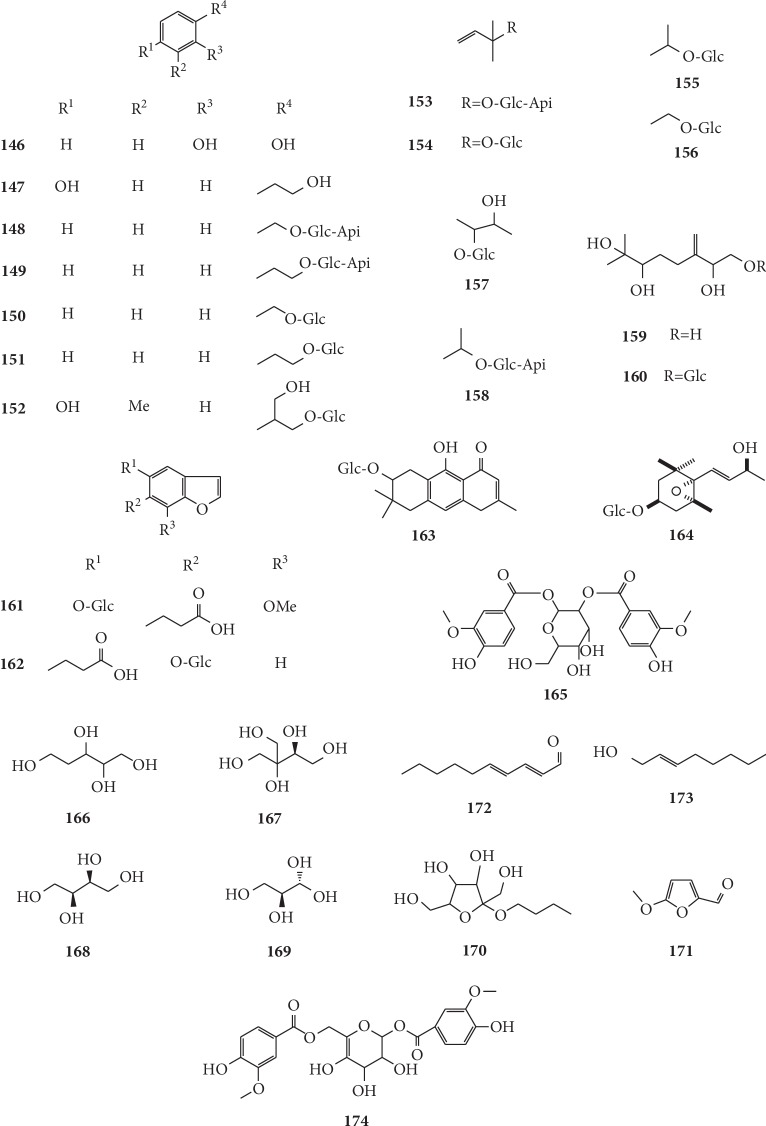
Structures of other chemical constituents in *G. littoralis.*

The identification of 18 trace elements in *G. littoralis* by Xu and Liu [77] via plasma emission spectrometry found that the contents of potassium, sodium, and phosphorus in peeled root samples of *G. littoralis* were significantly lower than those in nonpeeled root samples, which verified the enrichment of specific elements by root and root bark. The chemical structures of other chemical constituents isolated from *G. littoralis* are presented in Figure 13.

## 5. Pharmacological Activities

### 5.1. Immunoregulatory Activities

Yang et al. (2012) studied the effect of supercritical fluid extraction of carbon dioxide (SFE-CO_2_) extract from *G. littoralis* on T lymphocyte subpopulation induced by cytoxan (CTX) in immunosuppressed C57BL/6J mice. A globulimeter was used to detect WBC, RBC, and PLT, and the absolute number of T lymphocyte subpopulations was calculated by flow cytometry. The results showed that SFE-CO_2_ extract from *G. littoralis* increased the number of total CD3^+^ T cells as well as CD3^+^, CD4^+^ T cells, and CD3^+^, CD8^+^ T cells in the peripheral blood of immunosuppressed C57BL/6J mice (*P* < 0.05). However, no significant differences were observed in the ratio of CD3^+^CD4^+^/CD3^+^CD8^+^ T cells between the group treated with low doses of SFE-CO_2_ extract and the control group (*P* > 0.05), indicating that the low-dose SFE-CO_2_ extract restored the CD3^+^CD4^+^/CD3^+^CD8^+^ T-cell ratio in immunosuppressed mice to the normal level. The SFE-CO_2_ extract of *G. littoralis* Schmidt had a significant recovery effect on the peripheral immune system of immunosuppressed C57BL/6J mice [78].

Rong et al. prepared a mouse model of hyperthyroidism yin deficiency by administration of thyroxine and reserpine to mice, and the immunoregulation effect of the polysaccharide of Glehniae Radix (GLP) was applied to study weight fluctuation. The cytotoxic activity of NK cells, the T lymphocyte transformation function in the spleen, and the content of serum anti-sheep red blood cell antibodies IgM and IgG in yin deficiency mice were separately determined by MTT assays and indirect enzyme-linked immunosorbent assays. The results showed that GLP increased weight (*P* < 0.01, *P* < 0.05), significantly enhanced the killing activity of NK cells (*P* < 0.05) and T lymphocyte transformation function (*P* < 0.05), and increased the level of serum IgM and IgG antibodies (*P* < 0.05). It was suggested that GLP has the effect of nourishing yin and tonifying deficiency and could enhance the function of specific and nonspecific immunity [79].

Lv et al. established the immunosuppressed mouse model induced by cyclophosphamide and intragastrically administered different extracts (A: 1 g·mL^−1^ water extract, B: 1 g·mL^−1^ alcohol extract after water extraction, C: 1 g·mL^−1^ alcohol extract, and D: 1 g·mL^−1^ water extract after alcohol extraction) from the stems and leaves of *G. littoralis* at the dose of 2.34 g·kg^−1^ (the low-dose group) and 4.68 g·kg^−1^ (the high-dose group) once a day and continuously administered for 8 days. The blank control group, model control group, and American ginseng capsule 1.56 g·kg^−1^ group were intragastrically administered at the same time. The effects of cyclophosphamide on the phagocytic index of peripheral blood leukocytes, thymus gland, and spleen indices of mice were compared after the last administration. The results showed that the thymic index and spleen index of mice in the high-dose alcohol extract group were significantly increased, which suggests that the stem and leaf of Panax quinquefolium may inhibit the decrease in leukocyte count and thymic index in peripheral blood of mice induced by cyclophosphamide and enhance the phagocytic function of reticular endothelial system in immunocompromised mice [80].

### 5.2. Antitumor Activities

De la Cruz et al. conducted an assay on the survival ability of MCF-7 breast cancer cells treated with the aqueous extract of *G. littoralis* root. The results showed that *G. littoralis* extract decreased cell viability in a dose-dependent manner at concentrations of 50, 100, 200, and 400 *μ*g·mL^−1^. The reduction rate was 68.53%, 55.15%, 47.38%, and 39.57%, respectively. These data suggest that the extract has a strong antiproliferative effect on MCF-7 cancer cells, even at low concentrations. The results of flow cytometry showed that the aqueous extract of *G. littoralis* root can inhibit the proliferation of MCF-7 cells in the G0/G1 phase of cell cycle. Further, after 24 h of treatment, the expression of proteins associated with promoting cell cycle (CDK4 and cyclin D1) increased in a dose-dependent manner, while the expression of cycle-inhibiting proteins (P21 and P27) decreased. The aqueous extract of *G. littoralis* root inhibited the expression of CDK4 and cyclin D1 in the G1 phase by activating the protein kinases, P21 and P27, resulting in the inhibition of MCF-7 breast cancer cell proliferation [81].

Liu et al. studied the antitumor activity of different *G. littoralis* extracts *in vitro*. The three extractions, E1 (dissolved matter only in water), E2 (dissolved matter only in alcohol), and E3 (dissolved matter in water and alcohol, which was obtained by water from roots of *G. littoralis* and treated with alcohol), had *in vitro* pharmacological effect on lung cancer cell line (A549), stomach cancer cell line (SGC), and liver cancer cell line (HEP). The results showed that the different concentrations of the three extractions had certain inhibitory effect on liver cancer cell line (HEP) *in vitro*. There was no significant difference between the concentrations of E1 and E3. The inhibition rate of E2 was significantly higher than other two extractions when the concentrations at 300 *μ*g·mL^−1^. Most concentrations of the three extractions had anticancer activities against lung cancer cell line (A549). When the concentration of E3 was 37.5 *μ*g·mL^−1^, its inhibitory rate was significantly higher than that of the other two extractions. There was no significant difference among different concentrations of E1. When the two concentrations of E2 were 75 and 18.750 *μ*g·mL^−1^, their inhibitory rates were significantly higher than those of the other two extractions. The only concentration that had anticancer activities on stomach cancer cell line (SGC) was E2 at 300 *μ*g·mL^−1^, and its inhibitory rate was only 6.34% [82].

Um et al. found that the crude extract and the solvent-partitioned fractions (*n*-hexane, 85% MeOH, *n*-butanol, and water) of *G. littoralis* had significant synergistic inhibitory effect on the proliferation of HT-29 human colon cancer cells. The inhibitory rates of 50 *μ*g·mL^−1^ extract of *G. littoralis* on human colon cancer cells were 12%, 76%, 41%, 77%, and 86%, respectively, and the inhibitory effects were dose-dependent [69]. Dong et al. studied the anticancer activity of bergapten from *G. littoralis* extract. That study showed that 100 mg·L^−1^ bergapten had inhibitory effect on SGC-7901 and HEP-G2 gastric cancer cell lines [65].

### 5.3. Anti-Inflammatory Activities

The anti-inflammatory activities of methylene chloride fraction from *G. littoralis* extract (MCF-GLE) were studied by Yoon et al. (2010). MCF-GLE strongly inhibited the release of nitric oxide (NO), prostaglandin E2 (PGE2), tumor necrosis factor-*α* (TNF-*α*), and interleukin-1*β* (IL-1*β*) and significantly inhibited the mRNA and protein expression of inducible nitric oxide synthase and cyclooxygenase-2 (COX-2) in the RAW 264.7 macrophage cells by lipopolysaccharide stimulation in a dose-dependent manner. In addition, MCF-GLE inhibited nuclear factor kappa-B (NF-*κ*B) activation. The MCF-GLE also reduced the activation of extracellular signal-regulated kinase (ERK) and Jun kinase (JNK) in a dose-dependent manner. The anti-inflammatory properties of MCF-GLE were carried out by inhibiting the mRNA and protein expression of inducible nitric oxide synthase (iNOS) and COX-2, the expression of NO, PGE2, TNF-*α* and IL-1*β* in LPS-induced 264.7 macrophages cells. This study also found that the anti-inflammatory activity of MCF-GLE was mediated by inhibition of IXB-A phosphorylation, nuclear translocation of NF-*κ*B P65 subunit, and activation of MAPK (ERK and JNK) [2].

The effect of 70% ethanolic extract from *G. littoralis* (GLE) on inflammatory skin in mice was also studied by Yoon et al. (2010). Ear edema was induced by administering 12-*O*-tetradecanoyl-phorbol-13-acetate (TPA). The activities of IL-1, TNF-*α*, and myeloperoxidase (MPO), and histology in acute and chronic skin tissues were detected. At the same time, the vascular permeability test induced by acetic acid was carried out. 200 mg·kg^−1^ GLE significantly inhibited the topical edema of mouse ear, which led to a significant decrease in skin thickness, tissue weight, production of inflammatory cytokines, and activity of MPO mediated by neutrophils and polymorphonuclear leukocytes. In addition, GLE decreased significantly within the inflammatory site induced by chronic TPA and significantly inhibited vascular permeability induced by acetic acid in mice [83].

Other studies investigated the anti-inflammatory effect of imperatorin isolated from the root of *G. littoralis*. A mouse paw edema model was induced by lipopolysaccharide- (LPS-) stimulated mouse RAW264.7 macrophage cells and a carrageenan- (Carr-) induced mouse paw edema model. When RAW264.7 macrophages were treated with imperatorin together with LPS, the production of NO was significantly inhibited in a concentration-dependent manner. Western blotting showed that, in the LPS-induced RAW 264.7 macrophages, the expression of iNOS and COX-2 was blocked by imperatorin. At 4 h and 5 h after CARR administration, procyanidin decreased paw edema and the level of MDA and increased the activities of CAT, SOD, and GPX in paw edema. The Carr-induced iNOS and COX-2 expressions were also decreased, as well as the infiltration of neutrophils into the inflammatory site. Moreover, it could decrease the NO and tumor necrosis factor and prostaglandin E2 levels in serum [84].

### 5.4. Hepatoprotective Activities

Jin et al. observed the effect of the ethanol extract of *G. littoralis* (EEAR) on acute liver injury induced by CCl_4_ in rats. SD rats were divided into control group, CCl_4_ group, 150 mg·kg^−1^ EEAR + CCl_4_ group, 300 mg·kg^−1^ EEAR + CCl_4_ group, and 50 mg·kg^−1^ silibinin + CCl_4_ group, and the treatment was daily carried out with 0.5 mL extract by gastric administration for 7 days. The control group was injected intraperitoneally with saline, while the other groups were injected intraperitoneally with CCl_4_ (1 mL·kg^−1^) in the last day. And the levels of alanine aminotransferase (ALT), aminotransferase (AST), and alkaline phosphatase (ALP) in the serum were detected. The activities of superoxide dismutase (SOD) and catalase (CAT) and the level of malondialdehyde (MDA) of the liver homogenate were, respectively, detected by the yellow-terin oxidase method and the thiobarbituric acid method. Compared with the control group, the levels of ALT, AST, and ALP in the serum of CCl_4_ group were significantly increased (*P* < 0.05). Compared with CCl_4_ group, the content of AIT, AST, and ALP in the serum of the EEAR + CCl_4_ group decreased significantly (*P* < 0.05), the degree of HE staining was light, the activity of SOD and CAT in the cytoplasm was increased, and the level of MDA was decreased (*P* < 0.05). EEAR may significantly reduce inflammatory hepatocytes, improve steatosis of hepatocytes, and increase the number of normal hepatocytes [85].

Ultrastructural changes and apoptosis of tissue cells are the main manifestations of liver aging. The combined use of the root of *G. littoralis*, Radix Polygoni Multiflori, and Radix Salviae Miltiorrhizae in rats with liver diseases showed that these three Chinese medicines can not only significantly increase the level of IL-2 in serum, but also restore the volume of liver cells to the normal range, with homogenous nuclear chromatin. The number and morphology of mitochondria and rough endoplasmic reticulum returned to the normal range. The results indicated that the root of *G. littoralis*, Radix Polygoni Multiflori, and Radix Salviae Miltiorrhizae can be used to enhance the body's immunity, improve the ultrastructure of hepatocytes, inhibit the apoptosis of hepatocytes, and thus achieve antiaging purposes [86].

### 5.5. Others

#### 5.5.1. Antioxidant Activities

Oxidative stress is defined as the imbalance between the increase in reactive oxygen species concentration and the low activity of antioxidant mechanism. An increase in oxidative stress can result in damage to cellular structures, possibly damaging the tissues [87]. In a study examining the antioxidant ability of Shashen Maidong Decoction in a chronic bronchitis rat model, pharmacodynamic experiments were conducted by examining the activity of SOD, CAT, glutathione peroxidase (GSH-PX), and MDA as indices. The results showed that Shashen Maidong Decoction (with *G. littoralis* as the main component) increased the activity of SOD, CAT, and GSH-PX and decreased the MDA content in the serum of rats with chronic bronchitis [88]. An additional study showed that both the aqueous and organic extracts (500 *μ*g·mL^−1^) of *G. littoralis* have antioxidant properties. The aqueous extract of *G. littoralis* exhibited a strong inhibitory effect on hemolysis of rat erythrocytes. And the organic extract of the herb showed strong inhibition of lipid peroxidation in brain homogenates [89]. In the evaluation of microwave-assisted extraction of polysaccharides from *G. littoralis*, the herb exhibited high scavenging activity against 2,2-diphenyl-1-picryl-hydrazyl-hydrate (DPPH), hydroxyl, and superoxide anion radicals [90].

#### 5.5.2. Neuroprotective Activities

Park et al. subjected gerbils to transient global cerebral ischemia for 5 min. The extract of *G. littoralis* (GLE; 100 and 200 mg·kg^−1^) was taken once a day before conducting ischemic operations for 7 days. The neuroprotective effect was detected by immunohistochemistry and fluorescence staining, and the neuroprotective mechanism was detected by immunohistochemistry of superoxide dismutase (SOD)-1 and brain-derived neurotrophic factor (BDNF). The results showed that the ischemic injury area pretreated with 200 mg·kg^−1^ GLE protected pyramidal neurons (*P* < 0.05), and the activation of astrocytes (*P* < 0.05) and microglia (*P* < 0.05) in ischemic cornu ammonis 1 (CA1) area was significantly inhibited. In addition, GLE pretreatment significantly increased the expression of SOD-1 (*P* < 0.05) and BDNF (*P* < 0.05) in CA1 pyramidal cells of pseudo- and ischemic groups. It is suggested that GLE pretreatment can protect neurons from ischemic injury, and its neuroprotective mechanism may be closely related to the increase of SOD-1 and BDNF expression and the decrease of glial cell activation [91].

The effects of *G. littoralis* extract on the proliferation of hippocampal cells, the differentiation of neuroblasts, and the maturation of new neurons in adult mice were studied by Park et al. 5-Bromine 2-deoxyuridine (BrdU) was stained with double immunofluorescence staining for BrdU and neuron nuclear antigen. In addition, the expression of BDNF and its main receptor tropomyosin-related kinase B (TrkB) was detected by western blotting analysis. The extract of *G. littoralis* (200 mg·kg^−1^) significantly increased the protein levels of dentate gyrus (DG) and BrdUt/neuronal nuclear antigen (NeuN) cells in the subgranule area of DG (17.0 ± 1.5 cells/section) and doublecortin cells (72.0 ± 3.8 cells/sections) and significantly increased the protein levels of BDNF and TrkB (23.2% and 24.4% of the vehicle treatment group, respectively) [91].

#### 5.5.3. Antimicrobial Activities

The root of *G. littoralis* was extracted using methanol and was separated for the first time by methanol: chloroform silica gel column chromatography with different concentration ratios; 1,9-heptadecadiene-4,6-diyne-3,8,11-triol and 1,10-heptadecadiene-4,6-diyne-3,8,9-triol exhibited antibacterial and antifungal activities [67]. The leaves of *G. littoralis* were extracted with steam distillation and ether to obtain volatile oil components, which could then be separated to obtain *β*-pinene and *α*-pinene. *α*-Pinene, an essential oil from *G. littoralis*, exhibits better activity against *Trichophyton* than that exhibited by *β-*pinene and the total essential oil from *G. littoralis* [92]. Nineteen strains of endophytic fungi were obtained from *G. littoralis* by the plant tissue separation method using *Escherichia coli*, *Staphylococcus aureus*, and *Candida albicans* as indicator strains. Four strains of endophytic fungi had inhibitory effect against *Escherichia coli*, the ratio of the diameter of bacteriostatic circle of endophytic fungal fermentation concentrate to the diameter of 4 U·mL^−1^ gentamicin sulfate bacteriostatic circle (d/D) was 1.07, and 15 strains of endophytic fungi had inhibitory effect on *Staphylococcus aureus*, and the maximum d/D value was 0.65. Three strains of endophytic fungi had inhibitory effect on *Pseudomonas Albicans*. The maximum d/D value of endophytic fungal fermentation concentrate and 0.2 mg·mL^−1^ fluconazole was 1.27 [93].

In addition, Zhao study found that the water-soluble part had a certain antigastric ulcer effect and a certain scavenging effect of free radicals. It was previously reported that water-soluble part of *G. littoralis* has a good antilipid peroxide effect. The mechanism of this effect may be due to the promotion the free radical scavenging of gastric mucosa and reduced lipid peroxide [56]. The pharmacological activities of various bioactive ingredients in *G. littoralis* were presented in Table 3.

## 6. Toxicity

*G. littoralis* is considered a safe Chinese herbal medicine with beneficial effects in traditional use and modern pharmacology research. Modern toxicology research of *G. littoralis* is relatively rare, and research work is mainly concentrated in China.

Considering relevant historical books and records in China, the codecoction of Glehniae Radix and Radix et Rhizoma Veratri Nigri might produce toxicity or side effects [104]. Zhu et al. conducted a series of acute toxicity tests *in vivo* using uniform design method (two factors and seven levels) to investigate how the toxicity changed with different concentrations of these two drugs and whether decoction factors were correlated with toxicity. At the maximum Glehniae Radix dosage of 0.04 mL·g^−1^, no mice died. Another study found that the maximum dosage of the decoction of Glehniae Radix was 32 g·kg^−1^, which is 13.3-fold higher than that used in clinical practice, indicating that Glehniae Radix is almost nontoxic when it is used alone. However, in the toxicity study of different ratios of Glehniae Radix and Radix et Rhizoma Veratri Nigri decoction, the aqueous decoction of Radix et Rhizoma Veratri Nigri with the concentration of 2.566 g·kg^−1^ (the LD_50_ value in the 95% confidence interval is 2.566 g·kg^−1^), the codecoction, and mixed decoction of Glehniae Radix and Radix et Rhizoma Veratri Nigri with the proportion of 1 : 1 were administrated to the Kunming mice, respectively. The results showed the mortality rate of the mice administrated with mixed decoction of Glehniae Radix and Radix et Rhizoma Veratri Nigri was 25%, while the mortality rate of the codecoction of them was 65%. The toxicity of codecoction was higher than that of mixed decoction in the same dosage of Glehniae Radix and Radix et Rhizoma Veratri Nigri. In addition, when the ratio of Glehniae Radix and Radix et Rhizoma Veratri Nigri decoction increased from 1 : 1 to 1 : 4.19, the toxicity of their codecoction also increased, and the mortality of mice is reached to 90%. It is speculated that the promotion of the dissolution of the toxic component of Radix et Rhizoma Veratri Nigri in co-decoction may be the cause of the higher toxicity seen. Therefore, a prescription contained these drugs should be avoided in clinic practice [37].

Further study investigated the compatible effects of Glehniae Radix and Radix et Rhizoma Veratri Nigri decoction on cytochrome P450 isoenzyme activities in rat livers. The Wistar rats in the blank group were administered saline as a control, and the decoction of Radix et Rhizoma Veratri Nigri, the decoction of Glehniae Radix, and their codecoction were daily administrated at the dose of 0.081 g·mL^−1^, 1.08 g·mL^−1^, and 1.161 g·mL^−1^, respectively, for a week. The results showed that compared with the blank control group, the decoction of both Glehniae Radix and Radix et Rhizoma Veratri Nigri can induce CYP2C9 activities in rats, and their codecoction can significantly induce CYP1A2 and CYP2C9 activities. It is speculated that the induction effect of enzyme activity on the compatibility of the two drugs may accelerate the metabolic activation of the toxic components in the Radix et Rhizoma Veratri Nigri and enhance toxicity. Furthermore, compared with the decoction of Glehniae Radix, the CYP3A4 activity was significantly inhibited and the CYP1A2 activity was significantly induced, and there were no effects on the activities of CYP2C9 and CYP2C19 after administration of the codecoction of Glehniae Radix and Radix et Rhizoma Veratri Nigri in rats. It is suggested that when the codecoction of Glehniae Radix and Radix et Rhizoma Veratri Nigri was taken for a long time, the inhibition of CYP3A in rats changes the metabolism and physiological functions of endogenous substances and thus affects the metabolism of some of these components, resulting in toxicity. All these observations provide an experimental basis for the use of *G. littoralis* with *V. nigrum* [105].

To date, most of the toxicology research studies have focused on the roots of *G. littoralis*, and the toxicological investigations are in the basic stage. The aerial parts of this plant are sometimes used as aromatic vegetables, but the toxicity study has not been carried out, and further studies are needed on this herb.

## 7. Conservation Status and Proposals for *Glehnia* Species

The dried root of *G. littoralis* has been of great value in medicine (traditional medicine use, modern drug research, and development), as food ingredients (soup, porridge), and medicinal material health food (medicinal wine and drinkable medicinal tea), which has great commercial benefits. *G. littoralis* is one of the constructive species of the psammophyte community. It is suitable for growing on sandy beach or cultivating in fertile and loose sandy soil. According to peer-reviewed journals and field investigation, *G. littoralis* is distributed across the sandy seacoast, including China, Korea, Japan, and Russia and it plays an important role in coastal sand fixation and improvement saline-alkali soil [106, 107].

However, due to overdevelopment, industrialization, and the urbanization of beaches, as well as the overexploitation of *G. littoralis*, the wild resources of *G. littoralis* have been reduced gradually and the species has become increasingly endangered. Moreover, in the process of cultivation, as reported, the seeds of *G. littoralis* have the characteristics of being in deep dormancy with a high abortion rate. The production of wild *G. littoralis* has been on the verge of extinction and therefore could not fulfill the increasing market demand [108]. In this case, the cultivated varieties have become the main source of medicinal materials in the market. To ensure the quality of medicine, great attention is needed to protect germplasm resources, improve planting technology, and create suitable growing habitats for the species. Li et al. confirmed that the use of hormones combined with low temperature could significantly advance germination time and germination rate. This method was proven to be able to provide conditions for postmaturation of seed embryos or promote degradation of inhibiting germination substances to break dormancy and increase germination rate. It is beneficial to the artificial cultivation of *G. littoralis* [109].

Artificial breeding technology of *G. littoralis* is not only beneficial to the conservation and regeneration of endangered species, but also conducive to alleviation of sharp conflict between the production and demand of medicinal materials. Miao et al. used leaves and stalks as explant on *G. littoralis* and studied its callus-inducing medium. The experiments show that the best culture media for *G. littoralis* is 1/2MS + 0.4 mg·L^−1^ 6-BA + 1.5 mg·L^−1^ NAA. This condition can induce callus formation of *G. littoralis* with high efficiency and provide some theoretical data for the breeding of high-quality seedlings of *G. littoralis* [110]. Li et al. successfully established the clone of *G. littoralis* by using the tender stem, which proved the totipotency of the nonmeristematic tissue and cells of *G. littoralis*. This technology not only meets the needs of preservation of germplasm of *G. littoralis* but also ensures many seedlings in cultivation [111]. Song et al. used the method of sequence-related amplified polymorphism to analyze the genetic diversity of 80 individual materials of 8 populations from the genus Glehnia in China, thereby revealing why the germplasm of the herb can be adapted to the local living environment. This technology can be used to *G. littoralis* germplasm preservation to provide the scientific basis. As discussed before, these methods have broad application prospects in the protection and development of medicinal plant resources [112].

Nowadays, there are many methods to protect the endangered medicinal plants of *G. littoralis*, such as establishment of natural reserves, off-site protection and germplasm resources, improved breeding, and other ways to prevent the degradation, mixing, quality decline, and curative effect of the varieties of *G. littoralis*. To sustainably utilize *G. littoralis*, more focus needs to be on *G. littoralis* recovery, take acquisition and protection of inherit resources and intellectual property protection as important issues for wild *G. littoralis* habitat reservation. Transformation from using wild resources to utilizing artificial cultivating resources is a key to drive moderate exploitation of wild *G. littoralis* habitats and promote the standardization of its production process.

## 8. Conclusions and Perspectives

Available literature demonstrates that *G. littoralis* plays a vital role in TCM and nourishes yin, moistens the lung, expels phlegm, and arrests cough. In the past few decades, different classes of active components that possess multiple pharmacological properties have been reported to exist in *G. littoralis*. Further, modern clinical studies have evaluated the pharmacological activities of *G. littoralis*, which are related to its traditional uses. However, shortcomings remain in the utilization of *G. littoralis*.

Firstly, although it is a common medicine for treatment of lung and stomach diseases, the description of morphology of *G. littoralis* was not available in early ancient times. Therefore, it is unreasonable for some researchers to assume that the medicinal herbs referred to as *Shashen* are *Adenophora* plants, thus warranting further confirmation [20].

Secondly, in clinical application, the root of *G. littoralis* can be used in combination with a variety of medicinal materials to enhance its efficacy. For example, it combined with Aconiti Lateralis Radix Praeparata, Cinnamomi Ramulus, and Angelicae Sinensis Radix presents significant effect in the treatment of chronic arrhythmia. In addition, its combination with Astragali Radix, Hirudo, and Notoginseng Radix et Rhizoma is used to invigorate qi (the treatment for weakness) and activate blood circulation; it can also be used to treat apoplexy. However, the pharmacological action mechanisms underlying the synergistic effects of *G. littoralis* with these traditional Chinese medicinal materials are yet to be fully explained, and the other potential therapeutic effects remain unknown.

Thirdly, although *G. littoralis* is almost nontoxic, the dose should still be cautiously determined to avoid adverse reactions such as sensitization and irritation. In addition, its combined use with *V. nigrum* should be avoided. Furthermore, most studies on *G. littoralis* were mainly focused on its dried roots; thus, future investigations should be performed to evaluate the biological activities of its stems, leaves, and seeds.

Finally, specific studies exist on the cultivation, management, processing, and quality of *G. littoralis*; however, these are not systematic or comprehensive. Zhang studied the quality of *G. littoralis* using falcarindiol and panaxynol as the quality standards. The traditional technology of skin removal had significant negative effects on the content of the two active components in *G. littoralis* [113]. *G. littoralis* is often confused with *A. stricta* and other rhizomatous herbs. The identification method recorded in the Chinese Pharmacopoeia only contains characteristics of appearance and microscopic identification; therefore, it is necessary to establish an efficient and scientific method to ensure the safety of this medication [21]. Moreover, herb quality is affected by the geographical region; thus, it is necessary to standardize the management, processing, and quality of *G. littoralis* to adopt planting practices. Such efforts will lay a solid foundation for the development and utilization of Chinese medicinal resources and the modernization of Chinese medical material and increase the use of Chinese medical practices globally.

To summarize, this review provides an overview of past and current studies on the traditional uses, phytochemistry, pharmacological activities, and toxicity of *G. littoralis*. However, further studies exploring its efficacy, identifying its active components, and understanding its potential toxicity as a medicinal plant are warranted to ensure its safety for clinical applications. Moreover, it is necessary to conduct an in-depth study on the application of the other parts of this effective Chinese medicinal material.

## Figures and Tables

**Table 1:** Traditional uses of *G. littoralis* in China.

No.	Prescription name	Composition	Traditional uses	References
		Crude drug names (Latin names of original plants)		
1	Pinggan Yangfei Decoction	Paeoniae Radix Alba (*Paeonia lactiflora* Pall.) 9 g, Haliotidis Concha (*Haliotis diversicolor* Reeve; *Haliotis discus hannai* Ino; *Haliotis ovina* Gmelin; *Haliotis ruber* (Leach); *Haliotis asinina* Linnaeus; *Haliotis laevigata* (Donovan)) 15 g, fluoratum 12 g, Glehniae Radix (*Glehnia littoralis* Fr. Schmidt ex Miq) 9 g, Fritillariae Cirrhosae Bulbus (*Fritillaria Cirrhosa* D. Don; *Fritillaria unibracteata* Hsiao et K. C. Hsia; *Fritillaria przewalskii Maxim*.; *Fritillaria delavayi* Franch.; *Fritillaria taipaiensis* P. Y. Li; *Fritillaria unibracteata* Hsiao et K. C. Hsia var. *wabuensis* (S. Y. Tang et S. C. Yue) Z. D. Liu, S. Wang et S. C. Chen) 4.5 g, Mori Cortex (*Morus alba* L.) 9 g, Inulae Flos (*Inula japonica* Thunb.; *Inula britannica* L.) 9 g, Ruddle 12 g, Cyperi Rhizoma (*Cyperus rotundus* L.) 6 g, Lycii Fructus (*Lycium barbarum* L.) 9 g, Citri Exocarpium Rubrum (*Citrus reticulata* Blanco) 2.4 g, Armeniacae Semen Amarum (*Prunus armeniaca* L. var. *ansu* Maxim.; *Prunus sibirica* L.; *Prunus mandshurica* (Maxim.) Koehne; *Prunus armeniaca* L.) 9 g, Ostreae Concha (*Ostrea gigas* Thunberg; *Ostrea talienwhanensis* Crosse; *Ostrea rivularis* Gould) 15 g	Removing heat from the lungs, resolving phlegm, and relieving cough.	*Gaojing Zhizhi* (Yuan Dynasty, AD 1271∼1368)

2	Gejie Gujin Decoction	Rehmanniae Radix (*Rehmannia glutinosa* Libosch.) 18 g, Dioscoreae Rhizoma (*Dioscorea opposita* Thunb.) 9 g, Cordyceps (*Cordyceps sinensis* (BerK.) Sacc.) 9 g, Rubiae Radix Et Rhizoma (*Rubia cordifolia* L.) 6 g, Gecko (*Gekko gecko* Linnaeus) 4.5 g, Poria (*Poria cocos* (Schw.) Wolf) 9 g, Asini Corii Colla (*Equus asinm* L.) 9 g, Glehniae Radix (*Glehnia littoralis* Fr. Schmidt ex Miq) 9 g, Fritillariae Cirrhosae Bulbus (*Fritillaria Cirrhosa* D. Don; *Fritillaria unibracteata* Hsiao et K. C. Hsia; *Fritillaria przewalskii* Maxim.; *Fritillaria delavayi* Franch.; *Fritillaria taipaiensis* P. Y. Li; *Fritillaria unibracteata* Hsiao et K. C. Hsia var. *wabuensis* (S. Y. Tang et S. C. Yue) Z. D. Liu, S. Wang et S. C. Chen) 4.5 g, cristobalite 12 g, Ligustri Lucidi Fructus (*Ligustrum lucidum* Ait) 12 g	Curing deficiency of lung and kidney, wheezing cough, and phlegm blood	*Gaojing Zhizhi* (Yuan Dynasty, AD 1271∼1368)

3	Chagansaorilao-4 Decoction	Glehniae Radix (*Glehnia littoralis* Fr. Schmidt ex Miq), Glycyrrhizae Radix et Rhizoma (*Glycyrrhiza uralensis* Fisch.; *Glycyrrhiza inflata* Bat.; *Glycyrrhiza glabra* L.), Bistortae Rhizoma (*Polygonum historta* L.), Arnebiae Radix (*Arnebia euchroma* (Royle) Johnst.; *Arnebia guttata* Bunge)	Curing the infantile cough and clearing the heat	*Yifa Haijian* (compiled in 17th century)

4	Erdong ermu San	Asparagi Radix (*Asparagus cochinchinensis* (Lour-) Mern) 3 g, Ophiopogonis Radix (*Ophiopogon japonicus* (L. f) Ker-GawI.) 3 g, Anemarrhenae Rhizoma (*Anemarrhena asphodeloides* Bge) 3 g, Fritillariae Cirrhosae Bulbus (*Fritillaria Cirrhosa* D. Don; *Fritillaria unibracteata* Hsiao et K. C. Hsia; *Fritillaria przewalskii* Maxim.; *Fritillaria delavayi* Franch.; *Fritillaria taipaiensis* P. Y. Li; *Fritillaria unibracteata* Hsiao et K. C. Hsia var. *wabuensis* (S. Y. Tang et S. C. Yue) Z. D. Liu, S. Wang et S. C. Chen) 9 g, Adenophorae Radix (*Adenophora tetraphylla* (Thunb.) Fisch; *Adenophora stricta* Miq.) 9 g, Glehniae Radix (*Glehnia littoralis* Fr. Schmidt ex Miq) 9 g	Nourishing yin and resolving phlegm	*Chongding Tongsu Shanghan Lun* (Qing Dynasty, AD 1734∼1799)

5	Shenyan Maidong Decoction	Glehniae Radix (*Glehnia littoralis* Fr. Schmidt ex Miq) 9 g, Ophiopogonis Radix (*Ophiopogon japonicus* (L. f) Ker-GawI.) 9 g, cubilose 3 g, crystal sugar 12 g	Nourishing liver, replenishing qi and essence	*Chongding Tongsu Shanghan Lun* (Qing Dynasty, AD 1734∼1799)

6	Shenmai Ejiao Decoction	Glehniae Radix (*Glehnia littoralis* Fr. Schmidt ex Miq) 12 g, Ophiopogonis Radix (*Ophiopogon japonicus* (L. f) Ker-GawI.) 9 g, Asini Corii Colla (*Equus asinm* L.) 4.5 g, qi pi 3 g, Schisandrae Chinensis Fructus (*Schisandra chinensis* (Turcz.) Baill) 20 grains, glutinous rice 30 grains	Curing cold with blood, bleeding, and nourishing the lung	*Chongding Tongsu Shanghan Lun* (Qing Dynasty, AD 1734∼1799)

7	Yiguanjian Decoction	Glehniae Radix (*Glehnia littoralis* Fr. Schmidt ex Miq) 9 g, Ophiopogonis Radix (*Ophiopogon japonicus* (L. f) Ker-GawI.) 9 g, Angelicae Sinensis Radix (*Angelica sinensis* (Oliv.) Diels) 9 g, Rehmanniae Radix (*Rehmannia glutinosa* Libosch.) 18 g, Lycii Fructus (*Lycium barbarum* L.) 9 g, Toosendan Fructus (*Melia toosendan* Sieb. et Zucc.) 6 g	Nourishing yin and dispersing stagnated liver qi	*Xu Mingyi Leian* (Qing Dynasty, AD 1770)

8	Shashen Maidong Decoction	Glehniae Radix (*Glehnia littoralis* Fr. Schmidt ex Miq) 9 g, Polygonati Odorati Rhizoma (*Polygonatum odoratum* (MilL) Druce) 6 g, Glycyrrhizae Radix et Rhizoma (*Glycyrrhizae uralensis* Fisch.; *Glycyrrhizae inflata* Bat.; *Glycyrrhizae glabra* L.) 3 g, Mori Folium (*Morus alba* L.) 4.5 g, Ophiopogonis Radix (*Ophiopogon japonicus* (L. f) Ker-GawI.) 9 g, Lablab Semen Album (*Dolichos lablab* L.) 4.5 g, Trichosanthis Radix (*Trichosanthes kirilowii* Maxim; *Trichosanthes rosthornii* Harms) 4.5 g	Nourishing yin and lung disease	*Wenbing Tiaobian* (Qing Dynasty, AD 1798)

9	Haopi Siwu Decoction	Rehmanniae Radix (*Rehmannia glutinosa* Libosch.) 9 g, Glehniae Radix (*Glehnia littoralis* Fr. Schmidt ex Miq) 6 g, Angelicae Sinensis Radix (*Angelica sinensis* (Oliv.) Diels) 3 g, turtle shell 6 g, Paeoniae Radix Alba (*Paeonia lactiflora* Pall) 3 g, Artemisiae Annuae Herbe (A*rtemisia annua* L.) 3 g, Lycii Cortex (*Lycium chinense* Mill.; *Lycium barbarum* L.) 4.5 g, Moutan Cortex (*Paeonia suffruticosa* Andr.) 2.4 g, Glycyrrhizae Radix Et Rhizome (*Glycyrrhizae uralensis* Fisch.; *Glycyrrhizae inflata* Bat.; *Glycyrrhizae glabra* L.) 1.5 g	Nourishing yin and clearing heat, softening and resolving hard masses	*Bihua Yijing* (Qing Dynasty, AD 1808)

10	Liyin Hezhongjian Decoction	Rehmanniae Radix (*Rehmannia glutinosa* Libosch.) 9 g, Glehniae Radix (*Glehnia littoralis* Fr. Schmidt ex Miq) 9 g, Setariae Fructus Germinatus (*Setaria italica* (L.) Beauv.) 9 g, Lycii Cortex (*Lycium chinense* Mill.; *Lycium barbarum* L.) 6 g, Polygoni Multiflori Radix (*Polygonum multijiorum* Thunb.) 6 g, Artemisiae Annuae Herbe (*Artemisia annua* L.) 6 g, Hordei Fructus Germinatus (*Hordeum vulgare* L.) 6 g, Ludou Pi (*Glycine max* (L.) Merr.) 6 g, Ostreae Concha (*Ostrea gigas* Thunberg; *Ostrea talienwhanensis* Crosse; *Ostrea rivularis* Gould) 6 g, Paeoniae Radix Alba (*Paeonia lactiflora* Pall) 4.5 g, Crataegi Fructus (*Crataegus Pinnatifida* Bge. var. *major* N, E. Br; *Crataegus pinnatifida* Bge) 4.5 g, Magnoliae Officinalis Cortex (*Magnolia officinalis* Rehd. Et Wils.; *Magnolia officinalis* Rehd. et Wils. var. *Biloba* Rehd. et Wils) 3 g, Moutan Cortex (*Paeonia suffruticosa* Andr.) 3 g	Curing infantile malnutrition with deficiency of yin, stiff hair, dull skin, and crimson lips and tongues	*Bihua Yijing* (Qing Dynasty, AD 1808)

11	Jinshui Bawu Decoction	Glehniae Radix (*Glehnia littoralis* Fr. Schmidt ex Miq), Polygonati Odorati Rhizoma (*Polygonatum odoratum* (MilL) Druce), Dioscoreae Rhizoma (*Dioscorea opposita* Thunb.), Atractylodis Macrocephalae Rhizoma (*Atractylodes macrocephala* Koidz.), Astragali Radix (*Astragalus membranaceus* (Fisch.) Bge. var. *mongholicus* (Bge.) Hsiao; *Astragalus membranaceus* (Fisch.) Bge.), Lilii Bulbus (*Lilium lancifolium* Thunb.; *Lilium brownii* F. E. Brown var. *viridulum* Baker; *Lilium pumilum* DC), Longan Arillus (*Dimocarpus longan* Lour.), cubilose	Curing dry throat caused by impairment of qi	*Yimen Buyao* (Qing Dynasty, AD 1883)

12	Yinyang Qianyin Decoction	Rehmanniae Radix (*Rehmannia glutinosa* Libosch.) 30 g, Dendrobii Caulis (*Dendrobium nobile* Lindl; *Dctidrobium chrysotoxum* Lindl; *Dendrobium fimbriatum* Hook.) 9 g, Glehniae Radix (*Glehnia littoralis* Fr. Schmidt ex Miq) 9 g, Ophiopogonis Radix (*Ophiopogon japonicus* (L. f) Ker-GawI.) 9 g, Paeoniae Radix Alba (*Paeonia lactiflora* Pall) 9 g, Tortoise plastron 15 g, Dioscoreae Rhizoma (*Dioscorea opposita* Thunb.) 15 g, Poria (*Poria cocos* (Schw.) Wolf) 9 g	Curing pharyngeal ulcer and yin deficiency	*Waike Yijing* (Qing Dynasty, AD 1917)

13	Shashen Kuandong Decoction	Glehniae Radix (*Glehnia littoralis* Fr. Schmidt ex Miq) 6 g, Mori Cortex (*Morus alba* L.) 6 g, Asteris Radix Et Rhizoma (*Aster tataricus* L. f) 6 g, Farfarae Flos (*Tussilago farfara* L.) 9 g, Schisandrae Chinensis Fructus (*Schisandra chinensis* (Turcz.) Baill) 14 grains	Curing asthma caused by cough	*New book on family treatment* (compiled in 1929)

14	Gusui Wan	Bovine bone marrow 250 g, Ginseng Radix Et Rhizoma (*Panax ginseng* C. A. Mey.) 15 g, Rehmanniae Radix (*Rehmannia glutinosa* Libosch.) 30 g, Dragon Bone 30 g, Cervi cornus Colla 30 g, Cordyceps (*Cordyceps sinensis* (BerK.) Sacc.) 30 g, Polygoni Multiflori Radix (*Polygonum multijiorum* Thunb.) 30 g, Glehniae Radix (*Glehnia littoralis* Fr. Schmidt ex Miq) 30 g	Nourishing liver and kidney to supplement the essence and blood	*Jinfang Huiji* (compiled in 1959)

15	Jiawei Yiqi Liangxue Decoction	Rehmanniae Radix (*Rehmannia glutinosa* Libosch.) 12 g, Arnebiae Radix (*Arnebia euchroma* (Royle) Johnst.; *Arnebia guttata* Bunge) 9 g, Moutan Cortex (*Paeonia suffruticosa* Andr.) 6 g, Lycii Cortex (*Lycium chinense* Mill.; *Lycium barbarum* L.) 30 g, tortoise plastron 15 g, turtle shell 15 g, Adenophorae Radix (*Adenophora tetraphylla* (Thunb.) Fisch; *Adenophora stricta* Miq.) 9 g, Glehniae Radix (*Glehnia littoralis* Fr. Schmidt ex Miq) 9 g, Ophiopogonis Radix (*Ophiopogon japonicus* (L. f) Ker-GawI.) 9 g, Astragali Radix (*Astragalus membranaceus* (Fisch.) Bge. var. *mongholicus* (Bge.) Hsiao; *Astragalus membranaceus* (Fisch.) Bge.) 12 g, Codonopsis Radix (*Codonopsis pilosula* (Franch.) Nannf.; *Codonopsis pilosula* Nannf. var. *modesta* (Nannf.) L. T. Shen; *Codonopsis tangshen* Oliv.) 12 g, Atractylodis Macrocephalae Rhizoma (*Atractylodes macrocephala* Koidz.) 9 g, Dioscoreae Rhizoma (*Dioscorea opposita* Thunb.) 12 g	Nourishing yin and replenishing qi, removing heat from blood	*Xiashaonong Fang* (application in modern times)

16	Qingdu Lifei Decoction	Adenophorae Radix (*Adenophora tetraphylla* (Thunb.) Fisch; *Adenophora stricta* Miq.) 30 g, Glehniae Radix (*Glehnia littoralis* Fr. Schmidt ex Miq) 30 g, Asparagi Radix (*Asparagus cochinchinensis* (Lour-) Mern) 15 g, Persicae Semen (*Prunus persica* (L.) Batsch; *Prunus davidiana* (Carr.) Franch.) 9 g, Armeniacae Semen Amarum (*Prunus armeniaca* L. var. *ansu Maxim*.; *Prunus sibirica* L.; Prunus) 9 g, Fritillariae Cirrhosae Bulbus (*Fritillaria Cirrhosa* D. Don; *Fritillaria unibracteata* Hsiao et K. C. Hsia; *Fritillaria przewalskii* Maxim.; *Fritillaria delavayi* Franch.; *Fritillaria taipaiensis* P. Y. Li; *Fritillaria unibracteata* Hsiao et K. C. Hsia var. *wabuensis* (S. Y. Tang et S. C. Yue) Z. D. Liu, S. Wang et S. C. Chen) 9 g, Fritillariae Thunbergii Bulbus (*Fritillaria thunbergii* Miq) 9 g, Lycii Cortex (*Lycium chinense* Mill.; *Lycium barbarum* L.) 15 g, Prunellae Spica (*Prunella vulgaris* L.) 15 g, Gecko (*Gekko gecko* Linnaeus) 30 g, Trichosanthis Fructus (*Trichosanthes kirilowii* Maxim.; *Trichosanthes rosthornii* Harms) 30 g, Peucedani Radix (*Peucedanum praeruptorum* Durm) 9 g, Asteris Radix Et Rhizoma (*Aster tataricus* L. f) 12 g, Hedyotis Herb (*Hedyotis diffusa* Willd) 30 g, Scutellariae Barbatae Herba (*Scutellaria barbata* D, Don) 30 g, Paridis Rhizoma (*Paris polyphylla* Smith var. *yunnanensis* (Franch.) Hand. -Mazz.; *Paris polyphylla* Smith var. *chinensis* (Franch.) Hara) 30 g, Dendrobii Caulis (*Dendrobium nobile* Lindl.; *Dctidrobium chrysotoxum* Lindl; *Dendrobium fimbriatum* Hook.) 30 g	Moistening the lung and resolving phlegm, relieving poison, and removing blood stasis	*Prescription of Beijing traditional Chinese Medicine Hospital* (application in modern times)

**Table 2:** The chemical constituents isolated from *G. littoralis*.

Classes	Compound name	Part of plant	References
Phenylpropanoids	(*S*)-Phenyllactic acid (**1**)	Dried roots	[19]
	(*S*)-Phenyllactic acid methyl ester (**2**)	Dried roots	[19]
	Caffeic acid (**3**)	Dried root, dried underground parts	[38, 39]
	Chlorogenic acid (**4**)	Dried root, dried underground parts	[38, 39]
	Ferulic acid (**5**)	Dried root, dried underground parts	[38–40]
	3-Methoxy-4-*β*-D-glucopyranosyloxypropiophenone (**6**)	Dried underground parts, dried roots bark	[39, 41]
	4-[*β-*D-Apiofuranosyl-(1⟶6)-*β-*D-glucopyranosyloxy]-3-methoxypropiophenone (**7**)	Dried root, dried underground parts	[39, 42]
	2-Methoxy-4-(1-propionyl) phenyl *β*-D-glucopyranoside (**8**)	Fresh root and rhizome	[43]
	Syringin (**9**)	Dried root, dried roots bark	[41, 44]
	Glehnilate (**10**)	Dried underground parts	[45]

Coumarins	Umbelliferone (**11**)	Dried roots, dried roots and rhizomes	[46–48]
	Esculetin (**12**)	Dried root	[49]
	Umbelliferone 7-*O*-*β*-D-glucopyranoside (**13**)	Fresh fruit, dried root	[38, 50]
	Scopoletin (**14**)	Dried root and rhizome, dried root, dried underground parts	[38, 39, 46–48, 51, 52]
	Scopolin (**15**)	Dried root	[38, 49]
	Scopoletin-apiosyl-glucoside (**16**)	Dried root	[38]
	Osthenol 7-*O*-*β*-D-gentibioside (**17**)	Fresh fruit	[50]
	6-(3,3-Dimethylallyl)-7-hydroxycoumarin (**18**)	Dried root	[38]
	6-(3,3-Dimethylallyl)-7-hydroxycoumarin-apiosyl-glucoside (**19**)	Dried root	[38]
	6-(3,3-Dimethylallyl)-7-hydroxycoumarin-diglucoside (**20**)	Dried root	[38]
	7-*O*-(3,3-Dimethylallyl) scopoletin (**21**)	Dried root and rhizome	[52]
	(*S*)-Peucedanol 7-*O*-*β*-D-glucopyranoside (**22**)	Fresh roots and rhizomes	[53]
	(*S*)-Peucedanol 3′-*O*-*β*-D-glucopyranoside (**23**)	Fresh roots and rhizomes	[53]
	(*R*)-Peucedanol 3′-*O*-*β*-D-glucopyranoside (**24**)	Fresh roots and rhizomes	[53]
	(*S*)-7-*O*-Methylpeucedanol 3′-*O*-*β*-D-glucopyranoside (**25**)	Fresh roots and rhizomes	[53]
	(*S*)-Peucedanol 3′-*O-β-*D-apiofuranosyl-(1⟶6)-*β-*D-glucopyranoside (**26**)	Fresh roots and rhizomes	[53]
	(*S*)-7-*O*-Methylpeucedanol 3′-*O*-*β*-D-apiofuranosyl-(1⟶6)-*β*-D-glucopyranoside (**27**)	Fresh roots and rhizomes	[53]
	(+)-*cis*-(3′*S*,4′*S*)-Diisobutyrylkhellactone (**28**)	Whole plants	[54]
	3′-Senecioyl-4′-acetylkhellactone (**29**)	Whole plants	[54]
	3′-Isovaleryl-4′-acetylkhellactone (**30**)	Whole plants	[54]
	3′,4′-Disenecioylkhellactone (**31**)	Whole plants	[54]
	3′-Isovaleryl-4′-senecioylkhellactone (**32**)	Whole plants	[54]
	3′,4′-Diisobutyrylkhellactone (**33**)	Whole plants	[54]
	Marmesin (**34**)	Dried root and rhizome	[52]
	Rutaretin (**35**)	Dried root	[38]
	Marmesin 4′-*O-β-*D-apiofuranosyl-(1⟶6)-*β-*D-glucopyranoside (**36**)	Fresh roots and rhizomes	[53]
	Marmesinin (**37**)	Fresh and dried roots, dried underground parts, fresh fruit	[39, 50, 53]
	Leptophyllin (**38**)	Dried root	[38]
	(3′*R*)-Hydroxymarmesin 4′-*O*-*β*-D-glucopyranoside (**39**)	Fresh roots and rhizomes, dried roots and rhizomes, fresh fruit	[39, 50, 53]
	Oxymarmesinin 5′-*O*-*β*-D-glucopyranoside (**40**)	Fresh fruit	[50]
	Leptophyllin-2-glucoside (**41**)	Dried root	[21]
	Oxymarmesin 5′-*O-β*-D-glucopyranoside (**42**)	Fresh roots and rhizomes, dried underground parts, fresh fruit	[43, 50]
	Psoralen (**43**)	Dried root, dried underground parts, sliced roots, dried root and rhizome	[38, 39, 51, 52, 55]
	Xanthotoxol (**44**)	Dried root, dried underground parts, dried root and rhizome	[38, 39, 52]
	Xanthotoxin (**45**)	Dried root, sliced roots, dried root and rhizome	[38, 40, 42, 48, 51, 52, 55]
	Xanthotoxol 8-*O*-*β*-D-glucopyranoside (**46**)	Fresh fruit	[50]
	Xanthotoxol-diglucoside (**47**)	Dried root	[38]
	Xanthotoxol-triglucoside (**48**)	Dried root	[38]
	8-Methoxy-5-hydroxypsoralen (**49**)	Dried root	[38]
	Bergapten (**50**)	Dried root, dried root and rhizomes, sliced roots	[38, 40, 47, 48, 51, 52, 55]
	5-Methoxy-8-hydroxypsoralen (**51**)	Dried root	[38]
	Isoimpinellin (**52**)	Dried root	[38]
	8-Methoxy-5-hydroxypsoralen-glucopyranoside (**53**)	Dried root	[38]
	Bergaptol5-*O*-*β*-D-gentiobioside (**54**)	Dried root, dried underground parts	[39, 56]
	Imperatorin (**55**)	Dried root, sliced roots, dried root, and rhizomes	[38, 42, 51, 52, 55]
	8-(2-Hydroxy-3-methylbut-3-enoxy)-psoralen (**56**)	Dried root	[38]
	4″-Hydroxyimperatorin (**57**)	Dried root	[38]
	8-Geranyloxypsoralen (**58**)	Dried roots and rhizomes, fresh roots and rhizomes	[43, 52]
	4″-Hydroxyrmyperatorin 4″-*O-β-*D-glucopyranoside (**59**)	Fresh roots and rhizomes, dried underground parts	[39, 53]
	5″-Hydroxyimperatorin 5″-*O-β-*D-glucopyranoside (**60**)	Fresh roots and rhizomes	[53]
	Prangenin (**61**)	Dried root	[38]
	8-(1,1-Dimethylallyl)-5-hydroxypsorolen (**62**)	Dried root and rhizome	[52]
	Alloisoimperatorin (**63**)	Dried roots and rhizomes	[52]
	Byak-angelicol (**64**)	Dried root	[38]
	Byak-angelicin (**65**)	Dried root	[38]
	Phellopterin (**66**)	Dried root	[38, 57, 58]
	Isoimperatorin (**67**)	Dried root, dried underground parts, sliced roots, dried roots and rhizome	[38, 39, 42, 46, 51, 52, 55, 59]
	Cnidilin (**68**)	Dried root and rhizome, dried root	[38, 40, 52]
	Isooxypeucedanin (**69**)	Dried root	[38]
	Pabulenol (**70**)	Dried root	[38]
	Oxypeucedanin hydrate (**71**)	Dried root	[38]
	Oxypeucedanin (**72**)	Dried root	[38]
	8-[(2*E*,5*E*)-7-Hydroxy-3,7-dimethylocta-2,5-dieny-loxy] psoralen (**73**)	Fresh roots and rhizomes	[43]
	Bergaptin (**74**)	Dried roots and rhizomes	[52]
	8-[(2*E*)-6-Oxo-3,7-dimethyloct-2-enyloxy] psoralen (**75**)	Fresh roots and rhizomes	[43]
	Demethylsuberosin (**76**)	Sliced roots	[55]

Lignanoids	(-)-Secoisolariciresinol (**77**)	Dried underground parts	[39]
	(-)-Secoisolariciresinol 4-*O-β-*D-glucopyranoside (**78**)	Dried root, dried underground parts	[39, 49]
	Glehlinoside G (**79**)	Dried root	[64]
	Glehlinoside F (**80**)	Dried root	[61]
	Glehlinoside E (**81**)	Dried root	[61]
	Glehlinoside A (**82**)	Dried underground parts	[39]
	Glehlinoside B (**83**)	Dried underground parts	[39]
	Glehlinoside C (**84**)	Dried root, dried underground parts	[39, 49]
	Citrusin A (**85**)	Dried underground parts	[39, 62]
	Glehlinosides D (**86**)	Dried root	[63]
	Glehlinosides H (**87**)	Dried root	[64]
	3-Hydroxy-1-(4-hydroxy-3-methoxyphenyl)-2-[4-(3-hydroxy-1-(*E*)-propenyl)-2-methoxyphenoxy] propyl-*β*-D-glucopyranoside (**88**)	Dried root	[49]
	Glehlinosides I (**89**)	Dried root	[64]
	Glehlinoside J (**90**)	Dried root	[49, 64]
	2,3*E*-2,3-Dihydro-2-(3′-methoxy-4′-hydroxyphenyl)-3-hydroxymethyl-5-(3″-hydroxypropeyl)-7*-O-β*-D-glucopyranosyl-1-benzo[b] furan (**91**)	Dried root	[49]
	(7*R*,8*S*)-dehydrodiconiferylalcohol-4,9-di-*O*-*β*-D-glucopyranoside (**92**)	Dried root	[56, 60]

Flavonoids	Quercetin (**93**)	Dried underground parts	[39]
	Isoquercetin (**94**)	Dried underground parts	[39]
	Rutin (**95**)	Dried underground parts	[39]

Organic acids and derivatives	Salicylic acid (**96**)	Dried roots	[19, 40]
	Vanillic acid (**97**)	Dried root, dried underground parts	[38–40]
	4-*O*-*β*-D-Glucopyranosyl vanillic acid (**98**)	Dried root	[42]
	Vanillic acid 4-*O*-*β*-D-glucopyranoside (**99**)	Dried root	[44]
	Protocatechuic acid methyl ester (**100**)	Dried root	[49]
	1-*O*-Vanilloyl-*β*-D-glucose (**101**)	Dried root	[42]
	Vanillic acid 1-*O*-[*β*-D-apiofuranosyl-(1⟶6)-*β*-D-glucopyranoside] ester (**102**)	Dried root	[19, 42]
	Dibutyl phthalate (**103**)	Dried roots	[19]
	Tetracosanoic acid (**104**)	Dried roots of three-year-old	[65]
	9-Hydroxystearic acid (**105**)	Dried roots	[19]
	Glehlinosiden (**106**)	Dried roots bark	[41]
	Linoleic acid (**107**)	Dried root and rhizome	[51]
	Nonadecanoic acid (**108**)	Dried roots of three-year-old	[65]
	1-Linoloyl-3-palmitoylglycerol (**109**)	Dried roots	[46]

Terpenoids	(−)-Angelicoidenol [(2*R*,5*S*)-bornane-2,5-diol] 2-*O*-*β*-D-glucopyranoside (**110**)	Fresh roots and rhizomes	[66]
	(−)-Angelicoidenol 2-*O*-*β*-D-apiofuranosyl-(1⟶6)-*β*-*D*-glucopyranoside (**111**)	Fresh roots and rhizomes	[66]
	(2*R*,6*S*)-Bornane-2,6-diol 2-*O*-*β*-D-apiofuranosyl-(1⟶6)-*β*-D-glucopyranoside (**112**)	Fresh roots and rhizomes	[66]
	(4*R*)-*p*-Menth-1-ene-7,8-diol 8-*O*-*β*-D-glucopyranoside (**113**)	Fresh fruit	[50]
	(4*R*)-*p*-Menth-1-ene-7,8-diol 8-*O*-*β*-D-apiofuranosyl-(1⟶6)-*β*-D-glucopyranoside (**114**)	Fresh roots and rhizomes	[66]
	*trans*-*p*-Menth-2-ene-1,7,8-triol (**115**)	Fresh fruit	[50]
	*cis*-*p*-Menth-2-ene-1,7,8-triol (**116**)	Fresh fruit	[50]
	(2*R*)-Bornane-2,9-diol 2-*O*-*β*-D-apiofuranosyl-(1⟶6)-*β*-D-glucopyranoside (**117**)	Fresh roots and rhizomes	[66]
	(+)-Angelicoidenol [(2*S*,5*R*)-bornane-2,5-diol] 2-*O*-*β*-D-glucopyranoside (**118**)	Fresh roots and rhizomes	[66]
	*trans*-*p*-Menth-2-ene-1*α*,2*β*,8-triol (**119**)	Fresh fruit	[50]
	(4*S*)-*p*-Menth-1-ene-7,8-diol 8-*O*-*β*-D-apiofuranosyl-(1⟶6)-*β*-D-glucopyranoside (**120**)	Fresh roots and rhizomes	[66]
	(5*β*,10*α*)-Lasidiol angelate (**121**)	Dried root	[47]
	Botulin (**122**)	Dried root	[59]
	Lupeol (**123**)	Dried root	[59]

Polyacetylenes	Falcarindiol (**124**)	Dried root, sliced roots, dried root, and rhizome	[19, 42, 46, 51, 55, 67]
	(3*R*,9*Z*)-Heptadeca-1,9-dien-4,6-diyn-3-ol (**125**)	Dried roots	[19]
	(8*E*)-1,8-Heptadecadiene-4,6-diyne-3,10-diol (**126**)	Dried root, dried root and rhizome	[19, 40, 42, 51, 59]
	(3*S*,9*Z*)-Heptadeca-1,9-dien-4,6-diyn-3-ol (**127**)	Dried root, dried root and rhizome	[42, 46, 47, 51, 59]
	Ginsenoyne K (**128**)	Dried root	[42]
	(10*E*)1,10-Heptadecadiene-4,6-diyne-3,8,9-triol (**129**)	Dried root	[67]
	Falcaindiol (**130**)	Air- dried plant	[68, 69]
	Panaxydiol (**131**)	Air- dried plant	[69]
	(9*Z*)1,9-Heptadecadiene-4,6-diyne-3,8,11-triol (**132**)	Dried root	[67]

Steroids	*β*-Sitosterol (**133**)	Dried roots	[42, 46, 59]
	Daucosterol (**134**)	Dried root	[59]
	Stigmasterol (**135**)	Dried roots of three-year-old	[65]
	Cerevisterol (**136**)	Dried roots of three-year-old	[65]

Nitrogen compounds	(3*S*)-1,2,3,4-Tetrahydro-*β*-carboline-3-carboxylic acid (**137**)	Dried roots	[19]
	(1*S*,3*S*)-1-Methyl-1,2,3,4-tetrahydro-*β*-carboline-3-carboxylic acid (**138**)	Dried roots	[19]
	(1*R*,3*S*)-1-Methyl-1,2,3,4-tetrahydro-*β*-carboline-3-carboxylic acid (**139**)	Dried roots	[19]
	Uridine (**140**)	Dried underground parts	[39]
	Adenosine (**141**)	Dried root, dried underground parts, fresh fruit	[38, 39, 42, 50]
	9,9a-Dihydro-naphtho [2, 3-c] isoxazol-3(1H)-one (**142**)	Root	[70]
	Arginine (**143**)	Dried roots	[71]
	5′-Methylthioadenosine (**144**)	Dried roots	[72]
	L-Tryptophan (**145**)	Dried roots	[72]

Other chemical constituents	Catechol (**146**)	Dried roots	[19]
	4-Hydroxyphenethyl alcohol (**147**)	Dried roots	[19]
	Icariside F2 (**148**)	Dried root, fresh fruit	[42, 44, 50]
	Icariside D (**149**)	Dried root	[44]
	Benzyl *β*-D-glucopyranoside (**150**)	Fresh fruit	[50]
	Phenethyl *β*-D-glucopyranoside (**151**)	Fresh fruit	[50]
	1-*β*-D-Glucosyloxy-2-(3-methoxy-4-hydroxyphenyl) propane-1,3-diol (**152**)	Air-dried plant	[68]
	2-Methyl-3-buten-2-ol *β*-D-apiofuranosyl-(1⟶6)- *β*-D-glucopyranoside (**153**)	Fresh root and rhizome	[43]
	2-Methyl-3-buten-2-ol *β*-D-glucopyranoside (**154**)	Fresh fruit	[50]
	Isopropyl *β*-D-glucopyranoside (**155**)	Fresh fruit	[50]
	Ethyl *β*-D-glucopyranoside (**156**)	Fresh fruit	[50]
	Butane-2,3-diol 2-*O*-*β*-D-glucopyranoside (**157**)	Fresh fruit	[50]
	Isopropyl *β*-D-apiofranosyl-(1⟶6)-*β*-D-glucopyranoside (**158**)	Fresh fruit	[50]
	3,7-Dimethyloct-3(10)-ene-1,2,6,7-tetrol (**159**)	Fresh fruit	[50]
	(2*S*,6*Z*)-3,7-Di-methyloct-3(10)-ene-1,2,6,7-tetrol 1-*O*-*β*-D-glucopyranoside (**160**)	Fresh fruit	[50]
	6-Carboxyethyl-7-methoxy-5-hydroxybenzofuran 5-*O*-*β*-D-glucopyranoside (**161**)	Fresh fruit	[50]
	Cnidioside A (**162**)	Fresh root and rhizome	[43]
	*Sec-O*-glucosylhamaudol (**163**)	Dried root	[49]
	Corchoionoside A (**164**)	Fresh fruit	[50]
	1,2-Di-*O*-vanilloyl-*β*-D-glucopyranoside (**165**)	Dried roots	[5]
	2-Deoxy-D-ribitol (**166**)	Fresh fruit	[50]
	(3*R*)-2-Hydroxymethylbutane-1,2,3,4-tetrol (**167**)	Fresh fruit	[50]
	D-Threitol (**168**)	Fresh fruit	[50]
	Erythritol (**169**)	Fresh fruit	[50]
	*n*-Butyl *α*-D-fructofuranoside (**170**)	Dried root	[43]
	5-Methoxyfurfural (**171**)	Dried roots of three-year-old	[65]
	(2E,4E)-Deca-2,4-dienal (**172**)	Dried roots	[73]
	(E)-Oct-2-en-1-ol (**173**)	Dried roots	[73]
	1,6-Di-*O*-vanilloyl-*β*-D-glucopyranoside (**174**)	Dried roots	[5]

**Table 3:** Pharmacological activities of various bioactive ingredients in *G. littoralis.*

Pharmacological activity	Bioactive ingredient	Methods	Results and pathway	References
Immunoregulatory activity	Polysaccharide of Radix *G. littoralis.* (GLP)	The model of yin deficiency in mice was constructed with thyroxine and reserpine. High-dose group of GLP (800 mg·(kg·d)^−1^), medium-dose group of GLP (600 mg·(kg·d)^−1^), and low-dose group of GLP (400 mg·(kg·d)^−1^) are given by gavage for 6 consecutive days.	GLP obviously increased the weight of yin deficiency mice and also significantly raised the levels of antibody-forming cells in interperitoneal cavity (*P* < 0.01 or *P* < 0.05), and enhanced delayed-type hypersensitivity (DTH) reaction in mice (*P* < 0.01), but it had no effect on peritoneal macrophage coefficient and index.	[94]
	The SFE-CO_2_ extraction from Radix *G. littoralis*	Using cyclophosphamide to establish immunosuppressed mice model and the effects of the SFE extraction on it were studied. For 6 consecutive days, the intraperitoneal injection dose was 4 or 2g·kg^−1^·d^−1^ dissolved by DMSO with *Glehniae* SFE-CO_2_ extract.	The SFE extraction restored part of the immunological function in immunosuppressed mice. CD3^+^ T, CD3^+^ CD4^+^ T, and CD3^+^ CD8^+^ T in the low-dose group and the high-dose group were all higher than the immunity inhibition group, but lower than the normal control group (*P* < 0.05).	[95]
	The root of *G. littoralis*	Using cyclophosphamide to establish immunosuppressed mice model. For 10 consecutive days, the intraperitoneal injection dose was 4, 2, or 1 g·kg^−1^ with the root of *G. littoralis*.	*G. littoralis* can increase the weight of thymus gland and spleen, enhance the ability of mouse peritoneal macrophages to engulf neutral red, and improve the tumor killing rate of mouse lymphocytes and the killing ability of natural killer cells.	[96]
	Polysaccharides in *G. littoralis* Radix (GRP)	The mouse spleen lymphocytes were treated with 250, 125, 62.5, 31.25, 15.63, 7.8 mg·L^−1^ polysaccharide in the root of *G. littoralis*, and the three fractions (GRP-1, GRP-2, and GRP-3) were determined by MTT assay.	*In vitro* immunological activity results showed that the three polysaccharide components exhibited good immune activity.	[97]
	Extracts from leaves and stems of *G. littoralis*	The drug extracts from leaves and stems of *G. littoralis* were administered to the mice continuously for 15 days, and the intraperitoneal injection dose was 2.34 g·kg^−1^ or 0.59 g·kg^−1^. After 15 days, hemolytic value of mice was discussed.	Mice were given different doses of extracts from leaves and stems of *G. littoralis*. After 15 days compared with the control group, hemolytic value of mice in the water extract 2.34 g·kg^−1^ dose group, the alcohol extract 2.34 g·kg^−1^ dose group, and the American ginseng capsule group were significantly increased (*P* < 0.05). Extracts from leaves and stems of *G. littoralis* can regulate humoral immunity.	[68]

Antitumor activity	Crude extract and solvent-partitioned fraction of *G. littoralis*	Crude extract and solvent-partitioned fraction of *G. littoralis* were against human gastric cancer (AGS), HT1080 and U937 human cancer cells. The cells were treated with 5, 10, and 50 *μ*g·mL^−1^.	The crude extracts and solvent fractions dose-dependently inhibited cell proliferation. Especially, *n*-hexane and 85% aqueous MeOH fractions exhibited comparatively higher antiproliferative effects and reduced expressions of Bcl-2, COX-2, and iNOS genes.	[68]
	Polysaccharide from *G. littoralis* (PGL)	To investigate the anticancer activity of polysaccharide (PGL) from *G. littoralis* on human lung cancer cell line A549, after incubation, the cells were treated with various concentrations (380, 260, 160, 80, 40, and 20 *μ*g·mL^−1^) of PGL.	PGL could significantly reduce A549 cell proliferation in a time- and dose-dependent manner. In addition, PGL displayed an inhibitory activity for the A549 cells migration in Transwell migration assay. The results from both flow cytometry analysis and Hochst 3342 staining of apoptotic cells indicated that PGL could promote apoptosis and induce cycle arrest of A549 cells.	[98]
	*G. littoralis* crude drug slices	The *G. littoralis* crude drug slices were treated to human bronchial cell line16HBE and human lung cancer cell line H460 with 15 mg·mL^−1^, 10 mg·mL^−1^, and 5 mg·mL^−1^.	The extracts of *G. littoralis* from 5 mg·mL^−1^ to 15 mg·mL^−1^ had no toxic effect on 16HBE cells, significantly inhibited the proliferation and invasion ability of H460 cells, and upregulated the expression and secretion of TIMP2 in H460 cells. *G. littoralis* can upregulate the expression and secretion of TIMP2 in lung cancer cells and thus inhibit its migration and invasion.	[99]

Anti-inflammatory activity	Extract of the whole plants of *G. littoralis*	Purification of the *n*-hexane-soluble fraction of the whole plant of *G. littoralis* led to the isolation. All isolated compounds of *G. littoralis* from 0.5 to 50 *μ*M were evaluated by monitoring the inhibition of NO production in LPS-stimulated murine macrophage RAW 264.7 cells with aminoguanidine as the positive control (IC_50_ value: 16.6 *μ*M).	Extract of the whole plants of *G.littoralis* exhibited the significant inhibitory activity of the NO production with IC_50_ values ranging from 7.4 *μ*M to 44.3 *μ*M.	[54]

Antioxidant activity	*G. littoralis* ethyl acetate extract (GLEA)	Human hepatoma cell line HepG2 cells were treated to GLEA (50, 100, and 200 *μ*g·mL^−1^). Cell viability was evaluated by the MTT method. AST, ALT, and LDH production in a culture medium and intracellular MDA, GSH, and SOD levels were determined.	*G. littoralis* ethyl acetate extract significantly increased the relative cell viability by 7.11, 9.87, and 14.39%, respectively, and reduced the level of ALT by 10.39%, 34.27%, and 52.14%, AST by 9.89%, 15.16%, and 32.84%, as well as LDH by 15.86%, 22.98%, and 24.32% in culture medium, respectively. *G. littoralis* ethyl acetate extract could also remarkably decrease the level of MDA and increase the content of GSH and SOD in the HepG2 cells.	[100]

Neuroprotective activity	Ethyl acetate extract of *G. littoralis*	Prolyl oligopeptidase (POP) (50 *μ*l, 0.1 U·mL^−1^), Tris-HCl buffer (pH7.0, 840 *μ*l), and sample solution (10 *μ*L) saved 5 min at 30°C. Add 2 mmol·L^−1^ Z-G ly-Pro-pNA 100 *μ*l solution of 40%, 1-4-dioxide-hexacyclic ring, mix well, store 30 min, absorption was measured at 410 nm.	The inhibition rate of ethyl acetate extraction was 50%, which was similar to that of 1 mmol·L^−1^ proprolinal, the positive control, showing certain antipop enzyme effect.	[101]

Analgesic	The ethyl acetate fraction of the *G. littoralis* extract	The acetic acid-induced writhing test and the pentobarbital-induced hypnosis method were used to test for the analgesic effect at an oral dose of 1 g·kg^−1^.	The ethyl acetate fraction induced significant analgesia at an oral dose of 1 g·kg^−1^ and potently prolonged the sleeping time (>400% at 1 g·kg^−1^).	[51]

Inhibits fat accumulation	Extract of *G. littoralis* root hot water extract	To investigate the effects of *G. littoralis* root hot water extract on 3T3-L1 cell adipogenesis at dose of 400 *μ*g·mL^−1^, 200 *μ*g·mL^−1^, 100 *μ*g·mL^−1^, 50 *μ*g·mL^−1^ and in high-fat diet- (HFD-) induced obese mice. We measured intracellular lipid accumulation using oil red O staining *in vitro.* We also determined the expression levels of the adipogenesis-related proteins by RT-PCR and western blotting in HFD-induced obese mice.	The *G. littoralis* root hot water extract dose-dependently inhibited 3T3-L1 adipocyte differentiation and intracellular lipid accumulation in differentiated adipocytes. Body weight gain and fat accumulation were significantly lower in the GLE-treated HFD mice than in the untreated HFD mice. The GLE inhibits adipocyte differentiation and intracellular lipid accumulation by downregulating the adipogenic gene expression both *in vitro* and *in vivo*.	[102]

Hypolipidemic	Petroleum ether extract of the root of *G. littoralis*	To study the inhibitory effect of the root of *G. littoralis* petroleum ether part on TGF-*β*1-induced epithelial-mesenchymal transition in non-small-cell lung cancer A549, tested RT-qPCR at dose of 150 mg·L^−1^, 100 mg·L^−1^, and 50 mg·L^−1^.	The petroleum ether extract of Glehniae Radix could inhibit the growth of A549 cells in a concentration-dependent manner. The root of *G. littoralis* petroleum ether part group could effectively inhibit mRNA expressions of Col I, Vimentin, and *α*-SMA, but improve the expression of E-cadherin.	[103]

Antipeptic ulcer	The water-soluble portion of *G. littoralis* (petroleum ethyl acetate fraction)	The antigastric ulcer activity of H_2_0-soluble fraction was evaluated using the HCl/EtOH-induced gastric ulcer in mice. Then the water-soluble portion of *G. littoralis* from ethyl acetate fraction and petroleum of ethanol extract inject dose was 400 mg·mL^−1^ or 200 mg·mL^−1^, and the hydroxyl radical scavenging activity of H_2_O-soluble fraction was examined.	The results showed that the ulcer inhibition rate of the water-soluble part groups and the positive control group was 6.4%, 38.5%, and 60.8%, showing a certain antiulcer effect.	[56]

## Abbreviations

TCM: Traditional Chinese medicine
HPLC: High-performance liquid chromatography
GC-MS: Gas chromatography-mass spectrometry
SFE-CO_2_: Supercritical fluid extraction of carbon dioxide
CTX: Cytoxan
MTT: 3-(4,5-Dimethylthiazol-2-yl)-2,5-diphenyltetrazolium bromide
ELISA: Enzyme-linked immunosorbent assay
NO: Nitric oxide
PGE2: Prostaglandin E2
TNF*-α*: Tumor necrosis factor*-α*
IL-1*β*: Interleukin-1*β*
NF-*κ*B: Nuclear factor kappa*-*B
ERK: Extracellular signal-regulated kinase
JNK: Jun kinase
iNOS: Inducible nitric oxide synthase
COX-2: Cyclooxygenase-2
TPA: 12-*O*-tetradecanoyl-phorbol-13-acetate
MPO: Myeloperoxidase
LPS: Lipopolysaccharide
Carr: Carrageenan
ALT: Alanine aminotransferase
AST: Aminotransferase
ALP: Alkaline phosphatase
SOD: Superoxide dismutase
CAT: Catalase
MDA: Malodialdehyde
GSH-PX: Glutathione peroxidase
DPPH: 2,2-Diphenyl-1-picryl-hydrazyl-hydrate
BDNF: Brain-derived neurotrophic factor
CA1: Cornu ammonis 1
BrdU: 5-Bromine 2-deoxyuridine
TrkB: Tropomyosin-related kinase B
DG: Dentate gyrus
SGZ: Subgranular zone
AGS: Against human gastric cancer
Bcl-2: B-cell lymphoma 2
TIMP2: Tissue inhibitor of metalloproteinase 2
NeuN: Neuronal nuclear antigen
RT-qPCR: Real-time quantitative polymerase chain reaction
TGF: Transforming growth factor
*α*-SMA: *α*-Smooth muscle actin
POP: Prolyl oligopeptidase
HFD: High-fat diet.

## References

[1] Hiraoka N., Oyanagi M. (1991). Micropropagation of Glehnia (*Glehnia littoralis* Fr. Schmidt ex Miq.). *High-Tech and Micropropagation III*.

[2] Yoon T., Cheon M. S., Lee A. Y. (2010). Anti-inflammatory activity of methylene chloride fraction from Glehnia littoralis extract via suppression of NF-*κ*B and mitogen-activated protein kinase activity. *Journal of Pharmacological Sciences*.

[3] Miyazawa M., Kurose K., Itoh A., Hiraoka N. (2001). Comparison of the essential oils of Glehnia littoralis from northern and southern Japan. *Journal of Agricultural and Food Chemistry*.

[4] Lee S. C., Lee H. O., Kim K. (2015). The complete chloroplast genome sequence of the medicinal plant *Glehnia littoralis* F. Schmidt Ex Miq. (Apiaceae). *Mitochondrial DNA*.

[5] Gu X., Xu Y., Yuan Z. (2010). New divanilloylglucopyranoses from *Glehnia littoralis*. *Asian Journal of Traditional Medicines*.

[6] Committee for the Pharmacopoeia of PR China (2015). *Pharmacopoeia of PR China. Part I*.

[7] Jiangsu New Medical College (1977). *Chinese Materia Medica Dictionary*.

[8] Cui H. Y., Xu Y. P. (2009). Review of chemical constituents and pharmacological effects of *Glehnia littoralis*. *China Science and Technology Information*.

[9] Liu Y. F. (2006). *Glehnia littoralis* Fr. Schmidt ex Miq, a medicinal and edible plant. *Rural Practical Science and Technology Information (Modern Agriculture Research)*.

[10] Hiraoka N., Oyanagi M. (1988). *In vitro* propagation of *Glehnia littoralis* from shoot-tips. *Plant Cell Reports*.

[11] Yu D., Xu Z., Fujita H. (2019). Bibliometric analysis on the evolution of applied intelligence. *Applied Intelligence*.

[12] Yu D., Xu Z., Wang X. (2019). Bibliometric analysis of support vector machines research trend: a case study in China. *International Journal of Machine Learning and Cybernetics*.

[13] Yu D., Xu Z., Wang W. (2019). A bibliometric analysis of fuzzy optimization and decision making (2002-2017). *Fuzzy Optimization and Decision Making*.

[14] Yu D., Xu Z., Šaparauskas J. (2019). The evolution of “technological and economic development of economy”: a bibliometric analysis. *Technological and Economic Development of Economy*.

[15] Sun Y. F., Zhang X. S. (2015). Study development on pharmacological effect and clinical research of *Glehniae littoralis*. *Journal of Liaoning University of Traditional Chinese Medicine*.

[16] Chen W. N., Guo C. J., Shi J. Y. (2008). Review of the study development of Radix Glehniae in the latest ten years. *Qilu Pharmaceutical Affairs*.

[17] Geng Z. Y., Qiao Y., Yang X. Q. (2006). Research progress on *Glehniae littoralis*. *Modern Traditional Chinese Medicine*.

[18] Xin H., Yu L. (2008). Research progress in biology and chemical constituents of *Glehniae littoralis*. *Chinese Traditional and Herbal Drugs*.

[19] Zhang S., Cheng F., Yang L. (2019). Chemical constituents from *Glehnia littoralis* and their chemotaxonomic significance. *Natural Product Research*.

[20] Li B. G., Shi J. Y. (2002). A survey of the research on *Glehnia littoralis* in recent ten years. *Lishizhen Medicine and Materia Medica Research*.

[21] Li M., Chen Y. P., Han X. (2018). Research progress on Glehniae radix. *Food and Drug*.

[22] Institute of Botany and the Chinese Academy of Sciences (1992). *Flora of China 55*.

[23] Zhou S. R., Li B. L. (2008). Cultivation of *Glehnia littoralis*. *Special Economic Animal and Plant*.

[24] Tan C. Y., Wang M. L. (1997). Study on resource distribution and morphological characteristics of Laiyang Shashen. *Journal of Laiyang Agricultural College*.

[25] Xu B. H., Zhao H. P., Li J. (2017). Herbal textual research and original production area investigation of radix Glehniae. *China Pharmaceuticals*.

[26] Wu P. (1982). *Shen Nong’s Herbal Classic*.

[27] Zhang L. (1996). *Ben Cao Feng Yuan*.

[28] Su J. (1969). *New Xiu Materia Medica*.

[29] Li S. Z. (1975). *Compendium of Materia Medica*.

[30] Jia M. R., Zhang Y. (2016). *Dictionary of Chinese Ethnic Medicine*.

[31] Organization W. H., University S. N. (1998). *Medicinal Plants in the Republic of Korea: Information on 150 Commonly Used Medicinal Plants*.

[32] Liang H., Zhao G. (2005). An experimental study on the treatment of multiple sclerosis with Yiguanjian Decoction. *Pharmacology and Clinics of Chinese Materia Medica*.

[33] Ning B. B., Bian Y. Q., Zhang W. M. (2012). Study on liver protective effect of Yiguanjian Decoction. *Journal of Changchun University of Traditional Chinese Medicine*.

[34] Yang J. N., Zhou B. (2005). Effect on immunologic function of rats of yin deficiency treated by shashen Maidong decoction. *Journal of Practical Traditional Chinese Medicine*.

[35] Zhang J., Li Y. (2015). Progress of Shashen Maidong Decoction in treatment of non-small cell lung cancer. *Shandong Journal of Traditional Chinese Medicine*.

[36] Jie X., Bai X. F., Bai Y. H. (2017). Treatment of infantile cough with Mongolian medicine Chagansaorilao-4 decoction. *Journal of Medicine and Pharmacy of Chinese Minorities*.

[37] Zhu G. X., Wang Y. G., Li F. (2013). Research on the incompatibility of radix adenophorae, radix Glehniae combined with *Veratrum nigrum* L. By uniform design toxicity assay. *Chinese Journal of Integrated Traditional and Western Medicine*.

[38] Yang W., Feng C., Kong D. (2010). Simultaneous determination of 15 components in Radix Glehniae by high performance liquid chromatography-electrospray ionization tandem mass spectrometry. *Food Chemistry*.

[39] Yuan Z., Tezuka Y., Fan W., Kadota S., Li X. (2002). Constituents of the underground parts of *Glehnia littoralis*. *Chemical and Pharmaceutical Bulletin*.

[40] Yuan Z., Zhao M., Chen F. (2002). Chemical constituents from root and rhizome of *Glehnia littoralis*. *Chinese Traditional and Herbal Drugs*.

[41] Wang L. L. (2009). Study on Chemical Constituents and biological activity in the skin of *Glehnia littoralis*.

[42] Zhang J. (2014). A new aromatic glycoside from *Glehnia littoralis*. *Natural Product Research*.

[43] Kitajima J., Okamura C., Ishikawa T. (1998). New glycosides and furocoumarin from the *Glehnia littoralis* root and rhizoma. *Chemical and Pharmaceutical Bulletin*.

[44] Yuan Z., Zhou B. Y., Zhang Z. C. (2002). Glycosides from *Glehnia littoralis*. *Journal of Shenyang Pharmaceutical University*.

[45] Yuan Z., Kadota S., Xian L. (2002). Biphenyl ferulate from *Glehnia littoralis*. *Chinese Chemical Letters*.

[46] Su X., Li X. K., Tao H. X. (2013). Simultaneous isolation of seven compounds from *Glehnia littoralis* roots by off-line over pressured layer chromatography guided by a TLC antioxidant autographic assay. *Journal of Separation Science*.

[47] Um Y. R., Lee J. I., Lee J. H. (2010). Chemical constituents of the halophyte *Glehnia littoralis*. *Journal of the Korean Chemical Society*.

[48] Takuya K., Toshihiro S., Yusuke M. (2016). Comparative analysis of the constituents in Saposhnikoviae Radix and Glehniae Radix cum Rhizoma by monitoring inhibitory activity of nitric oxide production. *Journal of Natural Medicines*.

[49] Wang H., Xu Y., Yuan Z. (2011). Isolation and identification of chemical constituents of roots of *Glehnia littoralis*. *Journal of Shenyang Pharmaceutical University*.

[50] Ishikawa T., Sega Y., Kitajima J. (2001). Water-soluble constituents of *Glehnia littoralis* fruit. *Chemical and Pharmaceutical Bulletin*.

[51] Okuyama E., Hasegawa T., Matsushita T. (1998). Analgesic components of Glehnia root (*Glehnia littoralis*). *Natural Medicines*.

[52] Sasaki H., Taguchi H., Endo T., Yosioka I. (1980). The constituents of *Glehnia littoralis* Fr. Schmidt et Miq. structure of a new coumarin glycoside, osthenol-7-O-.BETA.-gentiobioside. *Chemical and Pharmaceutical Bulletin*.

[53] Kitajima J., Okamura C., Ishikawa T., Tanaka Y. (1998). Coumarin glycosides of Glehnia lifforalis root and rhizoma. *Chemical and Pharmaceutical Bulletin*.

[54] Lee J. W., Lee C., Jin Q. (2014). Pyranocoumarins from *Glehnia littoralis* inhibit the LPS-induced NO production in macrophage RAW 264.7 cells. *Bioorganic and Medicinal Chemistry Letters*.

[55] Masuda T., Takasugi M., Anetai M. (1998). Psoralen and other linear furanocoumarins as phytoalexins in *Glehnia littoralis*. *Phytochemistry*.

[56] Zhao Y. (2007). Studies on the Chemical constituents and Anti-gastric ulcer activity of *Glehnia littoralis*.

[57] Umetsu K., Kasahara M., Hiraoka N. (1992). Furanocoumarin composition in the fruit of *Glehnia littoralis* of different geographical origin. *The Japanese Society of Pharmacognosy*.

[58] Hiraoka N., Chang J.-I., Bohm L. R., Bohm B. A. (2002). Furanocoumarin and polyacetylenic compound composition of wild *Glehnia littoralis* in North America. *Biochemical Systematics and Ecology*.

[59] Zhang Y. B., Tang X. L., Li G. Q., Shi G. H. (2008). Study on Chemical Constituents of *Glehnia littoralis*. *Periodical of Ocean University of China*.

[60] Zhao Y., Kong W. X., Yuan Z. (2008). NMR characterization of a dihydrobenzofuran lignan glycoside. *Chinese Journal of Magnetic Resonance*.

[61] Kong W. X., Yuan Z. (2008). New lignan glycosides from *Glehnia littoralis*. *Chinese Chemical Letters*.

[62] Yuan Z., Zhou B. Y., Li X. (2002). NMR study of citrusin A. *Chinese Journal of Magnetic Resonance*.

[63] Wang L. L., Kong W. X., Yuan Z. (2008). A new 8-*O*-4′ neolignan from *Glehnia littoralis*. *Acta Pharmaceutica Sinica*.

[64] Xu Y., Gu X., Yuan Z. (2010). Lignan and neolignan glycosides from the roots of Glehnia littoralis. *Planta Medica*.

[65] Dong F., Liu H. Z., Sun Y. (2010). Isolation and identification of bergapten in dry root of *Glehnia littoralis* and preliminary determination of its antitumor activity *in vitro*. *Journal of Plant Resources and Environment*.

[66] Kitajima J., Okamura C., Ishikawa T., Tanaka Y. (1998). Monoterpenoid glycosides of *Glehnia littoralis* root and rhizoma. *Chemical and Pharmaceutical Bulletin*.

[67] Matsuura H., Saxena G., Farmer S., Hancock R., Towers G. (1996). Antibacterial and antifungal polyine compounds from Glehnia littoralis ssp. leiocarpa. *Planta Medica*.

[68] Kong C.-S., Um Y. R., Lee J. I., Kim Y. A., Yea S. S., Seo Y. (2010). Constituents isolated from *Glehnia littoralis* suppress proliferations of human cancer cells and MMP expression in HT1080 cells. *Food Chemistry*.

[69] Um Y. R., Kong C.-S., Lee J. I., Kim Y. A., Nam T. J., Seo Y. (2010). Evaluation of chemical constituents from *Glehnia littoralis* for antiproliferative activity against HT-29 human colon cancer cells. *Process Biochemistry*.

[70] Li G. Q., Zhang Y. B., Guan H. S. (2008). A new isoxazol from *Glehnia littoralis*. *Fitoterapia*.

[71] Huang L. L., Li M., Guo L. B. (2003). Determination of chemical components in *Glehnia littoralis*. *Chinese Journal of Misdiagnostics*.

[72] Zhao Y., Yuan Z. (2007). A new coumarin glycoside from *Glehnia littoralis*. *Acta Pharmaceutica Sinica*.

[73] Wang H. J., Wang L., Su B. Z., Yu Y. Z. (2010). Analysis of volatile constituents in root of *Glehnia littoralis* by GC-MS. *Qilu Pharmaceutical Affair*.

[74] Liu W., Li Z. Y., Tian Y. (2013). Research progress on chemical constituents and pharmacological effects of *Glehnia littoralis*. *International Journal of Pharmaceutical Research*.

[75] Liu Y.-H., Liu H.-Z., Xin H. (2010). Study on the content of coumarin in different parts of *Glehnia littoralis*. *Plant Science Journal*.

[76] Hua X., Wang H. X., Liu H. Z. (2009). Comparision on the content of coumarin in *Glehnia littoralis* during different harvest periods. *Acta Botanica Boreali-Occidentalia Sinica*.

[77] Xu D. C., Liu W. H. (1995). Analysis of trace elements in *Glehnia littoralis*. *Chinese Journal of Pharmaceutical Analysis*.

[78] Yang X. Y. (2012). The study on T lymphocyte subpopulation influence of *Glehnia littoralis* Schmidt on the C57BL/6J immunosuppressive mice. *Journal of Taishan Medical College*.

[79] Rong L. X., Lu S., Liu Y. M. (2013). Immunomodulatory effects of radix Glehniae polysaccharide on yin deficiency hyperthyroidism mice. *Chinese Journal of Basic Medicine in Traditional Chinese Medicine*.

[80] Lv F. J., Ye G. H., Xu Y. P. (2012). Effects of extracts from stems and leaves of *Glehnia littoralis* on immune function in immunosuppressed mice. *Lishizhen Medicine and Materia Medica Research*.

[81] De la Cruz J. F., Vergara E. J., Cho Y. (2015). *Glehnia littoralis* root extract induces G0/G1 phase cell cycle arrest in the MCF-7 human breast cancer cell line. *Asian Pacific Journal of Cancer Prevention Apjcp*.

[82] Liu X. L., Xin H., Tan L. L. (2009). Study on antitumor effect in vitro of different extractions by water from roots of *Glehnia littoralis*. *Journal of Anhui Agricultural Sciences*.

[83] Yoon T., Lee D. Y., Lee A. Y., Choi G., Choo B. K., Kim H. K. (2010). Anti-inflammatory effects of Glehnia littoralis extract in acute and chronic cutaneous inflammation. *Immunopharmacology and Immunotoxicology*.

[84] Huang G.-J., Deng J.-S., Liao J.-C. (2012). Inducible nitric oxide synthase and cyclooxygenase-2 participate in anti-inflammatory activity of imperatorin from *Glehnia littoralis*. *Journal of Agricultural and Food Chemistry*.

[85] Jin X., Zhen M. (2010). Protective effects of ethanol extract of *Glehnia littoralis* on carbon tetrachloride induced acute liver injury. *Journal of Changchun University of Traditional Chinese Medicine*.

[86] Bai Y., Zhou Z. Y., Zhang Y. L. (2007). Effects of traditional Chinese medicine on immune function and electron microscopic observation of hepatocytes in aging rats. *Journal of New Chinese Medicine*.

[87] Jean C. P. (2012). Oxidative stress. *Journal of Parenteral and Enteral Nutrition*.

[88] Li J., Li B. B., Yin L. (2013). Effects of shashen Maidong decoction on the antioxidant capacity of rats models with chronic bronchitis. *Journal of Traditional Chinese Medicine*.

[89] Ng T. B., Liu F., Wang H. X. (2004). The antioxidant effects of aqueous and organic extracts of *Panax quinquefolium, Panax notoginseng, Codonopsis pilosula, Pseudostellaria heterophylla* and *Glehnia littoralis*. *Journal of Ethnopharmacology*.

[90] Zhou H. Y., Lv S. (2016). Microwave-assisted extraction and antioxidant activity of polysaccharides from *Glehnia littoralis*. *Food Research and Development*.

[91] Park J. H., Shin B. N., Ahn J. H. (2018). Glehnia littoralis extract promotes neurogenesis in the hippocampal dentate gyrus of the adult mouse through increasing expressions of brain-derived neurotrophic factor and tropomyosin-related kinase B. *Chinese Medical Journal*.

[92] Shin S. (2005). Antifungal activities of essential oils from *Glehnia littoralis* alone and in combination with ketoconazole. *Natural Product Sciences*.

[93] Hou X. Q., Ren X. Y., Fu Y. J. (2015). Study on antibacterial activity and classification of endophytic fungi from *Glehnia littoralis*. *Chinese Traditional and Herbal Drugs*.

[94] Liu Y. M., Liu B., Wang J. F. (2005). The extraction on polysaccharide of Radix Glehniae and immuno-regulating effect on Yin deficiency mice. *Chinese Journal of Biochemical Pharmaceutics*.

[95] Feng L., Yang X. Y., Ji H. W. (2010). Effects of the SFE-CO2 extraction from Radix Glehniae on the immune functions of immune suppressed mice and chemical components analysis. *Chinese Journal of Hospital Pharmacy*.

[96] Li J. Y., Liu Y. Z., Zhang W. (2012). Study on the effect of Radix Glehniae on immune function in mice. *Chinese Journal of Laboratory Diagnosis*.

[97] Du B. X., Xiang M. R., Fu Y. P. (2018). Investigation of isolation, purification, structural identification and in vitro immunological function of polysaccharides in Glehniae radix. *Chinese Journal of Experimental Traditional Medical Formulae*.

[98] Wu J., Chen J., Song Z. (2018). Anticancer activity of polysaccharide from *Glehnia littoralis* on human lung cancer cell line A549. *International Journal of Biological Macromolecules*.

[99] Wang Z. F., Liu L., Liang L. (2018). Study on inhibition of migration and invasion of lung cancer cells by radix *Glehnia littoralis*. *Traditional Chinese Medicine Journal*.

[100] Kim B. H., Sun K. H., Kim S. P. (2017). In vitro protective effects of *Glehnia littoralis* on alpha-amanitin induced hepatotoxicity. *Journal of The Korean Society of Clinical Toxicology*.

[101] Li J. P., Yuan Z. (2005). Prolyl oligopeptidase inhibitory activity of *Glehnia littoralis* and isolation and identification of ferulic acid esters. *Chinese Herbal Medicine*.

[102] Hong H., dela Cruz J. F., Kim W. S., Yoo K., Hwang S. G. (2018). *Glehnia littoralis* root extract inhibits fat accumulation in 3T3-L1 cells and high-fat diet-induced obese mice by downregulating adipogenic gene expression. *Evidence-Based Complementary and Alternative Medicine*.

[103] Li Y. Q., Xiang M. R., Rong R. (2017). Inhibitory effect of Glehniae Radix petroleum ether part on TGF-*β*1-induced epithelial mesenchymal transition in A549 cells. *China Journal of Chinese Materia Medica*.

[104] Geng H. E. (2008). Textual research on the Chinese ginseng medicinal materials in the eighteen imcompatible pairs. *Journal of Practical Traditional Chinese Medicine*.

[105] Zhu G. X., Wang Y. G., Ma Z. C. (2013). Compatible effects of Radix Glehniae and Veratrum nigrum on cytochrome P450 enzymes activities in rat livers. *Pharmacology and Clinics of Chinese Materia Medica*.

[106] Zhang J. P. (2013). Comprehensive evaluation of land scape exploitation and application of wild herbaceous plant resources of Yuntai mountain. *Journal of Nanjing Forestry University (Natural Sciences Edition)*.

[107] Xu Z. F., Zhang Q. D., Li Q. D. (2006). Investigation and Analysis on the present situation of seed production and Seedling cultivation in the genuine producing area of *Glehnia littoralis*. *Journal of Shandong University of Traditional Chinese Medicine*.

[108] Fu L. G. (1992). *China Plant Red Date Book*.

[109] Li S., Lin B. C., Lv P. (2008). Tissue culture and establishment of a sexual line for *Glehnia littoralis* (A Gray) Fr. Schmidt ex Miq. *Natural Science Journal of Harbin Normal University*.

[110] Miao X. Y., He F. Q., Zhang X. M. (2014). Study on initiation of callus tissues and suspension cell culture of *Glehnia littoralis*. *Northern Horticulture*.

[111] Li H. B., Sun D., Huang C. Y. (2012). Study on plant regeneration from somatic embryos of vulnerable medicinal plant *Glehnia littoralis*. *China Journal of Chinese Materia Medica*.

[112] Song C. F., Liu Q. X., Zhou Y. F. (2014). Genetic diversity analysis of *Glehnia littoralis* (Apiaceae) revealed by SRAP. *Guihaia*.

[113] Zhang Y. B. (2007). Studies on quality control and evaluation technique of *Glehnia littoralis*.

